# Latent Profiles of Self-Reported Patient Privacy Protection Practices Among Hospital Healthcare Workers: Implications for Patient Safety and Interprofessional Education

**DOI:** 10.3390/healthcare14213623

**Published:** 2026-10-26

**Authors:** Yeonhu Lee

**Affiliations:** Department of Nursing, Honam University, Gwangju 62399, Republic of Korea; 2022092@honam.ac.kr; Tel.: +82-62-940-5552

**Keywords:** interprofessional education, privacy protection, information security, patient safety, healthcare professionals, latent profile analysis

## Abstract

**Highlights:**

**What are the main findings?**

- Healthcare workers’ self-reported patient privacy protection practices were summarized by three latent profiles: Low-practice (16.4%), Moderate-practice (29.1%), and High-practice (54.5%).
- About half of the respondents were consistently classified into the High-practice profile; lower practice was most evident in discussing patient information with unrelated staff and providing more than the minimum necessary information during consultations, and occupation was not associated with profile membership.

**What are the implications of the main findings?**

- Self-reported privacy practice varied mainly in overall level, and the apparent distinction between privacy- and security-related practice coincided with item wording (reverse- vs. positively worded items); the profiles offer a practical summary of this variation rather than discrete types.
- Behavior-specific interprofessional training and workflow supports (e.g., for minimum-necessary disclosure and record disposal) merit evaluation in intervention studies; targeting staff by demographic characteristics alone is not supported.

**Abstract:**

Background/Objectives: This study aimed to identify latent profiles of self-reported patient privacy protection practices among hospital healthcare workers and to explore factors associated with profile membership. Methods: In a secondary analysis of survey data from 213 staff at a national hospital, 11 practice items retained through Rasch-based screening were analyzed with latent profile analysis. Model selection used information criteria and a bootstrap likelihood ratio test; robustness was examined with alternative models, resampling, and sensitivity analyses. Associated factors were evaluated with classification error-corrected three-step multinomial logistic regression. Results: Three profiles were identified, Low-practice (16.4%), Moderate-practice (29.1%), and High-practice (54.5%), differing mainly in overall level. The Low-practice profile reported particularly low practice on items about discussing patient information with unrelated staff, minimum-necessary disclosure, and account sharing. The High-practice profile was stable across resamples, whereas the boundary between the lower profiles was not, and comparisons with continuous factor models did not establish discrete types. Respondents aged 50 years or older were more likely than those under 40 to belong to the Moderate-practice rather than the High-practice profile (odds ratio = 2.89; 95% confidence interval, 1.25–6.70), but the 95% interval from a full-procedure bootstrap, which accounts for profile-estimation uncertainty, included 1 (0.42–14.39), so this association is exploratory. Occupation was not associated with profile membership. Conclusions: Self-reported privacy practices varied mainly in overall level, and apparent differences between privacy- and security-related behaviors may partly reflect item wording. Healthcare organizations may consider behavior-specific, interprofessional training and workflow supports rather than defining at-risk groups by demographic or occupational factors; their effectiveness should be evaluated in intervention studies.

## 1. Introduction

### 1.1. Clinical Practice and Patient Privacy Protection

Healthcare workers, particularly nurses who maintain continuous contact with patients, repeatedly generate, access, and transmit sensitive patient information in the course of assessment and documentation, medication administration and treatment, handoffs, patient education, caregiver counseling, and interdisciplinary collaboration. Therefore, patient privacy protection goes beyond simple compliance with information security regulations and is connected to the core professional responsibility of putting confidentiality, patient advocacy, informational self-determination, therapeutic trust relationships, and patient safety into practice [1,2,3,4,5,6,7,8]. The spread of electronic medical records (EMRs), hospital information systems (HISs), cloud-based health information infrastructure, and artificial intelligence technologies has increased the accessibility and continuity of clinical information, but it has simultaneously amplified privacy protection risks such as unauthorized access, inappropriate disclosure, and insider misuse [9,10,11,12,13].

These risks are difficult to control with technical security measures alone and are largely shaped by clinical workflows and the everyday behaviors of the healthcare professionals who actually generate, access, transmit, and store information [11,13]. Nurses in particular handle patient information continuously across various locations and times of day and in interactions with multiple occupations and caregivers; they therefore require the competency to translate privacy protection principles into actual clinical judgment and behavior. Previous studies of nurses have reported that gaps may exist between awareness of privacy protection and actual performance, and that privacy protection awareness and performance may differ according to factors such as educational experience [1,2,3,4]. In addition, self-efficacy, social influence, nursing advocacy, privacy protection-related knowledge, and educational and environmental factors have been reported to be associated with privacy protection intentions or practices [5,6,8]. Meanwhile, studies on security behavior have reported that organizational support, the burden associated with policy compliance, system usability, and repeated security demands can affect policy compliance and security fatigue [14,15,16,17]. These findings indicate the need to understand patient privacy protection not only in terms of individual knowledge levels but also as the interaction between actual work behavior patterns and the clinical environment.

### 1.2. Limitations of Previous Research and the Need for a Person-Centered Approach

Existing privacy protection research has mainly used a variable-centered approach, calculating mean scores for knowledge, awareness, attitudes, or performance and examining their associations with demographic and job-related factors [1,2,3,4,5,6,7,8,10,11,14,15]. In nursing as well, studies identifying nurses’ levels of privacy awareness and practice and the factors influencing information protection behavior have accumulated [1,2,3,4,5,6,7,8], but most have focused on the average level of the entire group or on linear relationships among variables. Although such approaches are useful for identifying overall trends, nurses or healthcare workers with the same mean score may still differ in their combinations of lower-practice behaviors. For example, a person may perform well in system logout and password management yet be vulnerable to the verbal exposure of patient information or to providing more than the minimum necessary information during consultation, and the opposite behavior pattern is also possible. In clinical practice, what becomes the target of education and management may be such specific combinations of behaviors rather than the total score itself.

A person-centered approach focuses on individuals’ multivariate response patterns to estimate latent subgroups that share similar characteristics within a population. Latent profile analysis (LPA), which uses continuous indicators, is well suited to identifying group-specific practice patterns that are not revealed by mean levels alone [18,19,20,21,22]. If latent profiles can summarize privacy protection practice as several patterns, education can differentiate content according to the behaviors with lower practice, and management can target workflows and information management environments. However, the number of profiles should not be determined by a single information criterion alone; fit indices such as AIC and BIC, entropy and posterior probabilities, minimum group size, parsimony, and substantive interpretability should be considered jointly [18,19,20,21,22].

### 1.3. Distinctiveness from the Previous Study

In the author’s previous study [23], a diagnostic program and survey system were developed to enable healthcare institution staff to self-assess their privacy protection competency and receive educational materials on their areas of weakness. The previous study did not analyze the practice items as a separate measure. Before the present analysis, these items were screened with the Rasch rating scale model, and the 11 retained items were used as latent profile indicators (Section 2.2). Whereas the previous study focused on developing a diagnostic tool for privacy protection competency and an educational feedback system, this study poses an independent research question: using individual combinations of the selected practice indicators, it identifies “which lower-practice behaviors occur together.” In particular, person-centered analysis can reveal heterogeneity in self-reported practices involving patient information and provide evidence for designing tailored interprofessional education and organizational support strategies linked to handoffs, patient and caregiver communication, interdisciplinary collaboration, EMR use, and record management.

### 1.4. Study Objectives

- To identify the number and shape of the latent profiles of self-reported patient privacy protection practice using 11 practice indicators retained through Rasch-based screening, and to examine their robustness.
- To compare the item-level and Privacy/Security domain-level characteristics of the derived profiles, and to examine the profile distribution of participants, including nursing staff, and the behaviors with the lowest practice in each group.
- To explore factors associated with latent profile membership, focusing on age and occupation; to examine length of employment in a sensitivity analysis; and to interpret the results from the perspectives of patient safety, interprofessional education, and management.

## 2. Materials and Methods

### 2.1. Study Design and Participants

This study is a secondary data analysis that used existing cross-sectional survey data to explore the latent profiles of patient privacy protection practice and associated factors among healthcare institution staff, and to examine the implications of the results for practice and education. Participants were recruited through an institutional announcement to all 28 departments of National Hospital A between July and September 2024. Eligible participants were staff with direct or indirect access to patient information. Of the 217 staff invited, 213 (98.2%) consented and completed the questionnaire in the competency diagnosis program [23], and all reported experience in a patient-facing department. Participants included physicians, nurses, nursing assistants, administrative staff, medical technicians, social workers, pharmacy staff, clinical psychologists, and IT/technical and other support staff. Because the program accepted a submission only when all items had been answered, there were no missing data. The previous study excluded four respondents from its competency diagnosis because of inconsistent responding [23]; they were retained in the present analysis because post hoc exclusions can shape latent profile solutions, and the effect of excluding them was examined in a sensitivity analysis.

### 2.2. Indicators of Patient Privacy Protection Practice

The questionnaire, including an English version, is published as Table 1 of the previous study [23]. Its 18 practice items were screened with the Rasch rating scale model before the present analysis, following the principles presented by Bond and Fox [24]. Item fit was evaluated after excluding the 11 respondents with the poorest person fit, and items with an infit mean-square outside 0.6–1.4 and a standardized Z beyond ±2.0 were removed (seven items). For the 11 retained items, person reliability was 0.82 (separation 2.14) and item reliability was 0.96 (separation 5.03), and the four category thresholds were ordered, although the two lowest categories were rarely used. Item-level results are reported in Supplementary Table S1. In this study, patient privacy protection practice was defined as a higher-order concept encompassing the privacy (Privacy domain) and information security (Security domain) of patient information. For readers’ understanding and consistency in presenting the results, the 11 items were renumbered sequentially from Item 1 to Item 11, while the item content and scoring method of the previous study’s survey were retained. Each item was rated on a 5-point scale (1 = strongly disagree, 2 = disagree, 3 = neutral, 4 = agree, 5 = strongly agree) [23]; the items did not specify a recall period. Because Items 1, 2, 4, 5, and 8 asked about experiences of inappropriate behavior, they were reverse-coded using the 6 − raw score method so that higher scores on all items consistently indicated better patient privacy protection practice. Based on item content, the items were conceptually divided into Privacy, comprising six items on accessing, discussing, and providing patient information and sharing account information, and Security, comprising five items on passwords, logout, physical record storage, and disposal (Table 1). These indicators are also directly linked to the information access, information transfer, system use, and record management behaviors that are frequent in clinical practice. The questionnaire had no “not applicable” option; the possible influence of activities outside a respondent’s role was examined in sensitivity analyses.

### 2.3. General Characteristics and Factors Associated with Profile Membership

Considering sample sizes and job characteristics, occupation was reclassified into physicians, nursing staff (nurses and nursing assistants), administrative/support staff (administrative and IT/technical/other support), and other healthcare professionals (medical technology, social work, pharmacy, and clinical psychology). Age was categorized as under 40, 40–49, and 50 years or older, and length of employment as less than 5 years, 5 to less than 20 years, and 20 years or more. Experience working in patient-facing departments was excluded from the analysis of associated factors because all 213 participants had such experience. After age and length of employment were reclassified into the ordinal categories used in the analysis, Spearman’s rank correlation coefficient showed a strong positive correlation between the two variables (ρ = 0.786, *p* < 0.001). Accordingly, considering multicollinearity and possible redundancy in interpretation, age and occupation were included in the main model, and length of employment was examined separately in a sensitivity model in which it replaced age. Nursing staff was set as the reference category for occupation in the multinomial logistic regression.

### 2.4. Statistical Analysis

All statistical analyses were performed using Python (version 3.14.6; Python Software Foundation, Wilmington, DE, USA) in a Jupyter Notebook environment; all analyses were reproduced in File S1 with Python 3.13.16, NumPy 2.5.3, pandas 3.0.5, SciPy 1.18.1, scikit-learn 1.9.1, and Matplotlib 3.11.2 (Supplementary Table S7). NumPy and pandas were used for data preprocessing and matrix operations, and SciPy was used for numerical optimization, probability distribution calculations, and statistical testing. In addition, statsmodels was used to compute standard errors from the numerical Hessian matrix, scikit-learn was used for the adjusted Rand index and factor analysis, and Matplotlib was used to visualize the final results.

The core estimation procedures for the latent profile analysis and the classification error-corrected 3-step model were implemented directly in Python. Because all 11 practice indicators were measured on the same 1–5 range, they were analyzed without separate standardization, and reverse-coded items were recoded in advance so that higher scores indicated better privacy protection practice. The latent profile analysis (LPA) was performed based on a Gaussian finite-mixture model, and a maximum likelihood-based expectation-maximization (EM) algorithm was used for model estimation.

The item means of each profile were freely estimated, the variance of each item was constrained to be equal across profiles, and a class-invariant diagonal covariance structure was applied in which the covariances among indicators within a profile were fixed at 0 [18,19,20,21,22]. Models with one to five profiles were fitted sequentially. To reduce the influence of local maxima, multiple random starts were applied using both initial solutions based on quantiles of the total practice score and random initial solutions; 50 initial solutions each were used for the two- and three-profile models, and 80 each for the four- and five-profile models. Each model was additionally re-estimated from 400 random single starts to check whether the best log-likelihood was replicated. Up to 3000 EM iterations were allowed for each initial solution, with the convergence criterion for the change in log-likelihood set at 1 × 10^−9^, and among the solutions obtained for the same number of profiles, the admissible solution with the largest log-likelihood was selected as the final solution. Solutions in which the pooled within-profile variance of an indicator fell below 0.10 were regarded as spurious, a known artifact of fitting normal mixtures to discrete indicators [22]. The same criterion was applied in every analysis that re-estimated a latent profile model, including the bootstrap likelihood ratio test, bootstrap resampling, sensitivity analyses, and Monte Carlo simulations: in each fit, the admissible solution with the largest log-likelihood across starting values was retained, and fits without an admissible solution were excluded and counted (Supplementary Tables S2–S6).

Model selection comprehensively considered the Akaike information criterion (AIC), Bayesian information criterion (BIC), sample-size adjusted Bayesian information criterion (SA-BIC), entropy, average posterior probability (AvePP), minimum profile size, parsimony, and substantive interpretability [18,19,20,21,22,25,26]. Lower AIC, BIC, and SA-BIC values were interpreted as indicating relatively better model fit, and SA-BIC was calculated by applying (N + 2)/24 to adjust for sample size. Individual profile classification was based on each participant’s maximum posterior probability, and entropy and the AvePP of each profile were used to evaluate classification certainty under the fitted model, not to determine the number of profiles.

Factors associated with profile membership were analyzed using the Vermunt-type maximum-likelihood three-step approach, which corrects for classification error [27,28]. In the first step, an LPA without covariates was fitted to calculate each participant’s posterior probability for each profile; in the second step, participants were assigned to a single profile based on the maximum posterior probability. The posterior probabilities were then used to calculate the classification error matrix P(W=w|C=c) between the latent profiles and the profiles assigned by maximum posterior probability. In the third step, with these classification error probabilities fixed, a multinomial logistic model explaining latent profile membership probabilities according to covariates was estimated by maximum likelihood [29]. The Broyden–Fletcher–Goldfarb–Shanno (BFGS) algorithm was used for numerical optimization, and the standard errors of the regression coefficients were calculated from the numerical Hessian matrix at the optimum. Regression coefficients were exponentiated and presented as odds ratios (ORs); 95% confidence intervals (CIs) were calculated based on the normal approximation, and the significance of individual coefficients was evaluated with two-sided Wald tests. The High-practice profile was set as the reference profile for the outcome variable, and under 40 years (age) and nursing staff (occupation) were set as the reference categories. A main model entering age and occupation simultaneously was constructed; the significance of the overall model and of the age and occupation variable blocks was evaluated with likelihood-ratio tests against reduced models, and the explanatory power of the model was presented as McFadden pseudo-R^2^. All statistical tests were two-sided, and the significance level was set at 0.05.

The number of profiles was further evaluated with a parametric bootstrap likelihood ratio test (BLRT; 200 bootstrap samples per comparison) [30]; because non-normal indicators can favor additional classes [31], its results were interpreted together with the other criteria. Robustness was examined in four ways. First, the indicators were treated as ordered categorical variables in latent class models with class-specific category probabilities [32], with and without merging the rarely used lowest category with the next category. Second, the covariance assumptions were relaxed by allowing a residual covariance between Items 6 and 7, the only indicator pair with a within-profile residual correlation above 0.30 (a post hoc modification), and by allowing class-specific variances. Third, the profile models were compared with one- and two-factor models and with a factor mixture model [33]; all of these Gaussian models were fitted by maximum likelihood to the same 213 respondents and 11 indicators, with log-likelihoods that included all normalizing constants, so that their information criteria were on the same scale. Fourth, stability was assessed with nonparametric bootstrap resampling (200 resamples), in which profiles were labeled by ordering them by mean item score, as in the main analysis (checked against optimal matching of the profile means to the reference solution), and agreement between classifications was quantified with the adjusted Rand index (ARI) [34].

Sensitivity analyses (a) separated registered nurses and nursing assistants, (b) restricted the sample to clinical staff, (c) excluded the four respondents removed in the previous study [23], (d) excluded 11 respondents with invariant responses across all indicators or with the lowest response to Item 3, which may not apply to non-clinical roles, and (e) added the screened-out item on accessing other patients’ records, whose removal was close to the cut-off in a replication of the screening. Uncertainty in the covariate model was assessed with a bootstrap of the full three-step procedure (200 resamples), excluding resamples in which a profile had fewer than five members. Instead of a post hoc power calculation [35], Monte Carlo simulations based on the fitted model examined the recovery of the profile structure (100 simulated samples) and the power to detect the observed age association (200 simulated samples). All analyses can be reproduced with the code in Supplementary File S1, in which all random seeds are fixed.

### 2.5. Ethical Considerations and Use of Secondary Data

Data collection was approved by the Institutional Review Board of Honam University (IRB No. 1041223-202402-HR-41), and all participants provided written consent to participate in the study [23]. This study is a secondary analysis of research data collected in a previous study that developed a privacy protection diagnostic program [23]. The previous study [23] described the development of a 47-item competency diagnosis program combining awareness and practice items and reported each participant’s lowest-scoring processing stage. It did not analyze the practice items as a separate measure and included no item screening, profile analysis, or covariate analysis. The present study used the practice indicators retained through the Rasch-based screening to identify latent profiles, explore associated factors, and interpret the patterns from the perspectives of patient safety and education.

## 3. Results

### 3.1. General Characteristics of Participants

Of the total 213 participants, 20 (9.4%) were physicians, 115 (54.0%) were nursing staff, 43 (20.2%) were administrative/support staff, and 35 (16.4%) were other healthcare professionals, with nursing staff accounting for the largest proportion. By age, 81 (38.0%) were under 40 years, 50 (23.5%) were 40–49 years, and 82 (38.5%) were 50 years or older; by length of employment, 71 (33.3%) had less than 5 years, 75 (35.2%) had 5 to less than 20 years, and 67 (31.5%) had 20 years or more. The descriptive distribution of the 115 nursing staff by profile was 24 (20.9%) in the Low-practice profile, 36 (31.3%) in the Moderate-practice profile, and 55 (47.8%) in the High-practice profile (Table 2). Thus, 52.2% of nursing staff belonged to profiles other than the High-practice profile; however, the statistical association between occupation and profile membership was evaluated separately in the 3-step analysis in Section 3.5.

### 3.2. Determining the Number of Latent Profiles

Latent profile models with one to five profiles were fitted sequentially. The information criteria decreased as the number of profiles increased, but the smallest groups in the 4-profile and 5-profile models contained only 2 (0.9%) and 5 (2.3%) participants, respectively; these were excessively small, suggesting possible over-extraction and unstable classification. In contrast, the 3-profile model showed high classification certainty (entropy = 0.915, AvePP = 0.955–0.971), and its smallest group retained an adequate size of 35 participants (16.4%). Accordingly, considering not only the information criteria but also classification certainty, minimum group size, parsimony, and substantive interpretability, the 3-profile model was selected as the final model (Table 3). The BLRT favored three over two profiles (likelihood ratio [LR] = 114.6, *p* < 0.01) and also four over three profiles (LR = 103.1, *p* < 0.01); however, the four-profile solution reproduced the lowest profile exactly and separated only two respondents with atypical response patterns. Higher-likelihood four- and five-profile solutions found with additional random starts were spurious (Supplementary Table S2). The two-profile solution merged the lowest profile with most of the Moderate-practice profile (53 of 62 respondents).

### 3.3. Characteristics of the Latent Profiles

**Figure 1:**
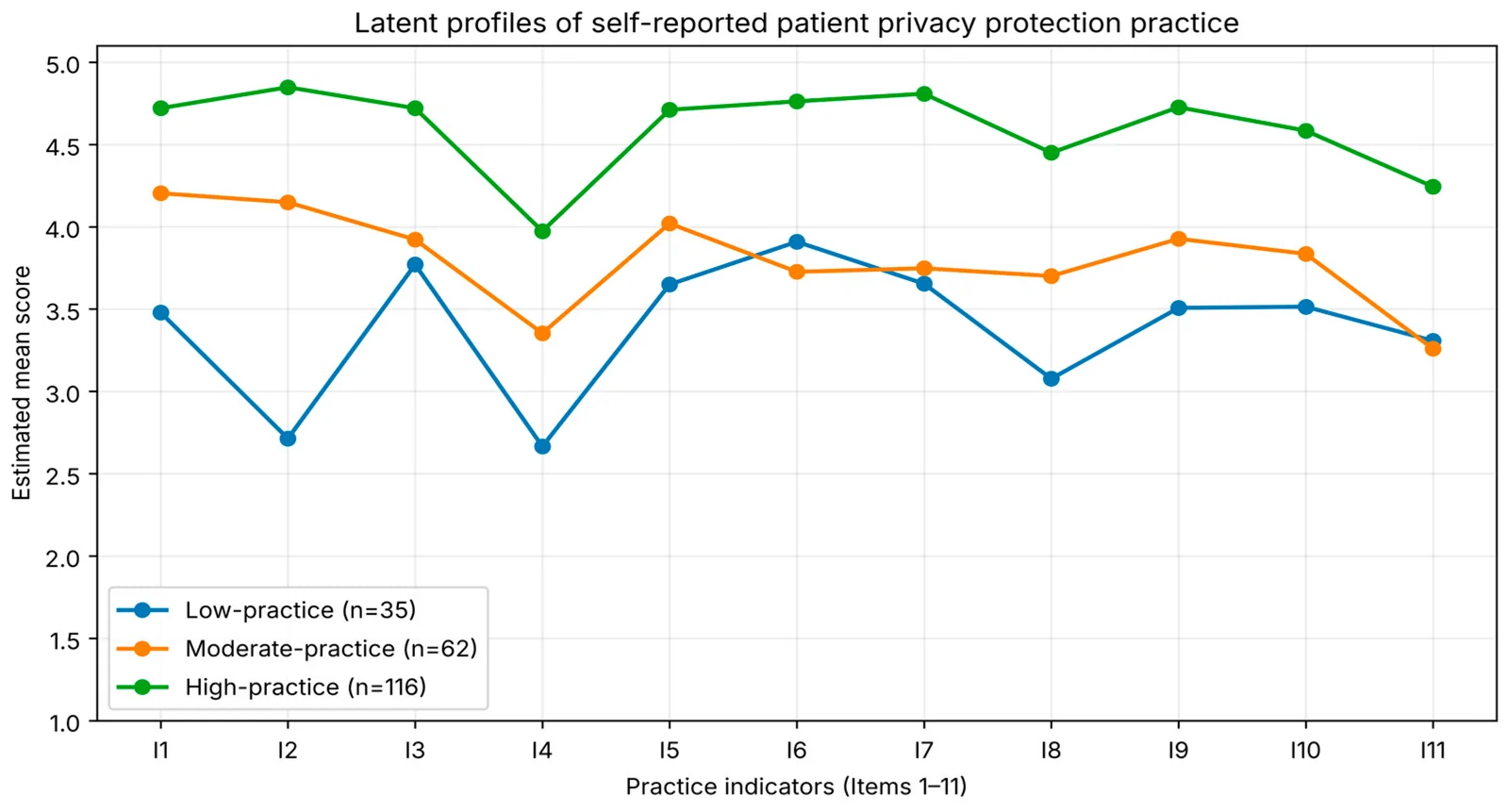
Latent profiles of self-reported patient privacy protection practice.

Based on item and domain means, the final three profiles were named the Low-practice profile, the Moderate-practice profile, and the High-practice profile (Table 4, Figure 1).

The Low-practice profile comprised 35 participants (16.4%), with a Privacy mean of 3.23 and a Security mean of 3.58. Self-reported practice was particularly low for not discussing patient conditions with unrelated staff (Item 2, M = 2.71), providing only the minimum information necessary during consultation or referral (Item 4, M = 2.67), and not sharing medical information system IDs/passwords with colleagues (Item 8, M = 3.08). All three items were behaviors related to discussing or providing clinical information or to using system accounts.

The Moderate-practice profile comprised 62 participants (29.1%), with a Privacy mean of 3.89 and a Security mean of 3.70. Appropriate disposal of records past their retention period (Item 11, M = 3.26) and provision of minimum necessary information (Item 4, M = 3.35) were relatively low. The High-practice profile comprised 116 participants (54.5%), with a Privacy mean of 4.57 and a Security mean of 4.62; however, even in this group, provision of minimum necessary information (Item 4, M = 3.97) and disposal of records past their retention period (Item 11, M = 4.24) were lower than the other items. In other words, these two behaviors, which involve judging the scope of information provision and managing the record life cycle, may represent organization-wide areas for improvement regardless of profile level.

### 3.4. Robustness and Stability of the Profiles

Ordinal latent class models also had the lowest BIC for three classes, although these classes differed mainly in overall level and agreed only modestly with the profile classification (ARI = 0.32); because their likelihood is defined for discrete responses, their BIC values were not compared with those of the Gaussian models. Models with class-specific variances produced boundary solutions with variances at the computational lower bound of 0.05, which were inadmissible under the 0.10 criterion. The remaining models were Gaussian models fitted to the same respondents and indicators with the same likelihood conventions (Supplementary Table S3). Compared with the reported three-profile model, which has a diagonal covariance structure (BIC = 5733.1), a single-factor model (BIC = 5723.5) and a two-factor model (BIC = 5676.1) fitted better. The two factors largely separated the five reverse-worded privacy items from the positively worded security items, and the only positively worded privacy item (Item 3) loaded weakly on both; because item content and wording direction coincide, the factors may partly reflect item wording rather than distinct privacy- and security-related practice. A separate comparison concerns the three-profile model that allowed a residual covariance between Items 6 and 7, which was added after inspection of residual correlations: it yielded a similar solution (ARI = 0.72) and a slightly lower BIC than the two-factor model (5666.8 vs. 5676.1). Thus, the profile model fitted worse than the two-factor model under the diagonal covariance assumption and slightly better after one post hoc residual covariance was added; neither comparison establishes discrete underlying types.

All 200 bootstrap resamples yielded an admissible three-profile solution. The High-practice profile was consistently recovered (median 53.8% of respondents; 95% interval 39.5–61.1%), whereas the division between the two lower profiles varied (median ARI = 0.70), and the lower privacy- than security-related scores of the lowest profile were reproduced in 45.5% of resamples (70.5% when profiles were matched to the reference profiles by their means). Labeling the profiles by mean item score agreed with optimal matching of the profile means in 140 of 200 resamples; the results for the High-practice profile were identical under either labeling, whereas those for the two lower profiles were not (Supplementary Table S4). The three-profile solution was reproduced when the four respondents excluded in the previous study were removed (ARI = 0.98), when the analysis was restricted to clinical staff (ARI = 0.96), when 11 respondents with invariant or potentially non-applicable responses were removed (ARI = 0.97), and when the screened-out item was added (ARI = 0.98). Monte Carlo simulations indicated that a three-profile structure of the observed form can be recovered with N = 213 when selection is restricted to admissible solutions: with responses rounded to the 1–5 format, BIC selected three profiles in 92 of the 100 simulated samples (Supplementary Tables S4 and S5).

### 3.5. Factors Associated with Latent Profile Membership

In the classification error-corrected 3-step multinomial logistic regression model with the High-practice profile as the reference, the overall model entering age and occupation simultaneously was significant (likelihood-ratio χ^2^ = 23.72, df = 10, *p* = 0.008; McFadden pseudo-R^2^ = 0.056). The overall association of age was significant (likelihood-ratio *p* = 0.008), whereas the overall association of occupation, with nursing staff as the reference category, was not significant (*p* = 0.141). Compared with those under 40 years, participants aged 50 years or older were more likely to belong to the Moderate-practice than to the High-practice profile (OR = 2.89, 95% CI 1.25–6.70, *p* = 0.013). In contrast, the odds of Moderate-practice profile membership for those aged 40–49 years and the age associations with the Low-practice profile were not significant. In a bootstrap of the full three-step procedure (160 of 200 resamples analyzed), the age ≥ 50 association was in the same direction in 87% of resamples, but its 95% percentile interval was wide and included 1 (0.42–14.39; 0.45–7.62 when profiles were matched to the reference solution by their means; Supplementary Table S6); this association is therefore regarded as exploratory.

For other healthcare professionals, an individual contrast indicating lower odds of Low-practice profile membership was observed (OR = 0.17, *p* = 0.033); however, because the overall occupation block was not significant, it was not interpreted (Table 5). In the 3-step sensitivity model entering length of employment instead of age, the length of employment block was significant (likelihood-ratio *p* = 0.027), but none of the individual odds ratios (ORs) for each category was significant at the 0.05 level (Supplementary Table S6). Given the strong correlation between age and length of employment, it is difficult to clearly separate the independent associations of these two characteristics in the current sample. When registered nurses and nursing assistants were entered as separate categories, the distinction did not improve the model (LR χ^2^ = 3.98, df = 2, *p* = 0.137), and the age association was similar (OR = 2.60, 95% CI 1.10–6.14; Supplementary Table S5).

## 4. Discussion

To understand patient privacy protection practice beyond mean scores, this study reanalyzed 11 self-reported practice items retained through Rasch-based screening from a person-centered perspective. Three profiles were identified: Low-practice, Moderate-practice, and High-practice profiles. Nursing staff made up 54.0% of the sample, and the measured behaviors included information management activities common to many professions, such as discussing patient information, providing information during consultation, account management, logout, and record disposal. In this respect, the results merit interpretation in terms of interprofessional education, management, and patient safety. However, because the overall association of occupation was not significant, the derived profiles should not be regarded as types unique to nursing staff.

### 4.1. Interpretation of the Profiles and Their Lower-Practice Behaviors

The three profiles are best read as a person-centered summary of variation, mainly in overall level, rather than as discrete types. A two-factor model fitted better than the profile model with a diagonal covariance structure, and a profile model with one post hoc residual covariance fitted only slightly better than the two-factor model, so the data do not distinguish discrete profiles from continuous dimensions. The two factors largely separated reverse-worded from positively worded items; because five of the six privacy items were reverse-worded and all security items were positively worded, the apparent distinction between privacy- and security-related practice cannot be separated from item wording in these data. The High-practice profile, comprising about half of the respondents, was robust across resamples and sensitivity analyses, whereas the division between the two lower profiles was not stable. In the full sample, the Low-practice profile reported particularly low practice on Items 2, 4, and 8, but this pattern was not consistently reproduced in resampling and should be confirmed in other samples.

The Low-practice profile, accounting for 16.4% of the total, reported lower practice in Privacy behaviors, such as accessing, discussing, and providing patient information, than in system management behaviors. In particular, scores were low for not discussing patient conditions with unrelated staff, providing only the minimum necessary scope of information during consultation, and not sharing account information. These three behaviors relate to handoffs, conversations involving sensitive information in multi-bed rooms, responding to inquiries from caregivers, interdisciplinary consultation, and EMR use. Because five of the six privacy indicators were reverse-scored items asking about past inappropriate behavior, this profile is best described as comprising respondents who more often reported such behavior. Previous studies of nurses have also reported gaps between privacy awareness and performance and the importance of education [1,2,3,4], and self-efficacy and social influence have been shown to be associated with information protection intentions or behaviors [5,6,8]. Therefore, rather than being taught only as an abstract legal compliance obligation, privacy protection needs to be addressed as a competency to judge and practice, in actual clinical situations, “to whom, what information, to what extent, and in what manner to communicate.”

The Moderate-practice profile showed intermediate levels in both Privacy and Security but relatively low practice in providing minimum necessary information and record disposal, and even in the High-practice profile, the same two items were lower than the other behaviors. This suggests that if information protection education focuses only on system security behaviors such as changing passwords, logging in and out, and locking screens, gaps may remain at the stages of providing patient information and managing the record life cycle. Because nurses and other clinicians repeatedly transmit patient information during handoffs, interdepartmental consultation, coordination of tests and treatments, discharge education, and caregiver counseling, the principle of minimum necessary information needs to be linked to clinical judgment. In addition, standard operating procedures and organizational support systems should be established so that the management of temporary notes, printouts, and records past their retention period does not depend solely on individual judgment [9,10,11,12,14,15]. These commonly low-scoring items are better interpreted as organization-level indicators that call for joint improvement of workflows and system design than as grounds for blaming specific individuals.

Items 4 and 11 were the two lowest-scoring items in the sample as a whole and in the Moderate- and High-practice profiles. Item 4 asked whether respondents provide as much personal information as possible when referring a patient (reverse-scored). Clinicians may regard comprehensive information as good practice for continuity of care, so low scores may reflect a tension between clinical norms and the minimum-necessary principle rather than a lack of knowledge alone. Consistent with this, scores were lower among nursing staff (3.41) and physicians (3.50) than among administrative and support staff (3.77). Item 11 concerns disposing of records after their retention period or asking the responsible department to do so. In many hospitals, this task belongs to medical records units, so low scores may reflect limited opportunity or unclear role allocation. Both behaviors may therefore respond to workflow supports, such as referral templates that specify the minimum necessary data and centralized disposal procedures, as well as to training. This hypothesis requires testing.

### 4.2. Implications for Interprofessional Education and Management

These findings suggest that privacy protection education could move away from delivering identical content to all staff members toward differentiating educational content and support methods according to the behaviors with lower practice, an approach that requires evaluation. For the Low-practice profile, case- and simulation-based education using actual clinical situations, such as unnecessary information exposure during handoffs, sensitive conversations in multi-bed rooms, inquiries from caregivers or acquaintances, the scope of information provision during consultation, and account sharing among colleagues, may be appropriate. For the Moderate-practice profile, repeatedly checking and providing feedback on the principle of minimum necessary information and record disposal procedures within workflows may be useful, whereas the High-practice profile may benefit from periodic reinforcement of lower-scoring items and maintenance of organizational standards. Such an approach could make privacy protection education more practice-centered and interprofessional, integrating patient advocacy, professional ethics, patient safety, and informatics competencies; whether it improves practice or patient safety remains to be tested.

Nurse managers and other unit managers can examine the actual information management environment, including how handoffs are conducted, the communication environment in multi-bed rooms, EMR screen exposure, the management of printouts and temporary notes, account-sharing practices, and role-based access privileges. Educators could link privacy protection principles to clinical cases in orientation and in-service education for all professions and evaluate behavior change rather than merely checking knowledge after education [5,6,7,8].

### 4.3. Age- and Occupation-Related Factors

In the main three-step model, age was associated with profile membership, and participants aged 50 years or older were more likely than those under 40 years to belong to the Moderate-practice profile relative to the High-practice profile. However, the bootstrap interval that incorporated profile-estimation uncertainty included 1, power was modest, and the cross-sectional design does not allow causal inference; this association is therefore exploratory and requires confirmation. In addition, because the same age association was not observed between the Low-practice profile and the High-practice profile, it is difficult to interpret this as a simple linear relationship in which “the older the person, the lower the privacy protection practice.” Furthermore, because this study did not directly measure digital literacy, technology acceptance, workload, security fatigue, or system usability, the mechanism of the age association cannot be conclusively attributed to these variables. As previous research has suggested that security fatigue and compliance costs can affect security behavior [16,17], future research needs to measure EMR experience, digital competency, system usability, educational methods, and the burden of repetitive security procedures, rather than age itself, and verify their relationships with profile membership.

The overall association of occupation was not significant. Therefore, the descriptive distribution in Table 2, in which 52.2% of nursing staff belonged to profiles other than the High-practice profile, should not be interpreted as relatively lower practice among nursing staff or as a risk inherent to the occupation. Rather, privacy protection practice patterns may be more directly related to the actual frequency of access to patient information, responsibility for generating and transmitting information, intensity of EMR use, and the work environment than to simple occupational classifications such as physicians, nursing staff, and administrative/support staff. From a nursing perspective, these results indicate the need for research that specifically measures the information access, transmission, and storage behaviors repeated in nursing work processes, rather than approaches that use occupation itself as a risk marker. Registered nurses and nursing assistants differ in education, legal responsibility, and EMR access. In a sensitivity analysis, nursing assistants were less often in the High-practice profile than registered nurses (37.2% vs. 54.2%), but the difference was not statistically significant (Supplementary Table S5). Future studies need to distinguish between nurses and nursing assistants and include work unit, EMR access level, frequency of generating and accessing patient information, digital competency, security culture, and workload to determine whether heterogeneous practice patterns within nursing staff are replicated.

### 4.4. Limitations

This study has several limitations. First, because cross-sectional data from a single national hospital were used, causal relationships cannot be inferred, and generalization to healthcare institutions of other types and regions and to other nursing settings is limited. Second, because all practice indicators were based on self-reported surveys rather than observed behavior, social desirability bias may exist. In addition, the items did not specify a recall period, and five of the six privacy indicators were reverse-scored items about past inappropriate behavior, so the contrast between privacy- and security-related practice may partly reflect item wording. Third, a Gaussian LPA treating 5-point items as continuous was applied. Ordinal latent class models also favored three classes, but class membership depended on the model, and a continuous two-factor model fitted better than the profile model with a diagonal covariance structure and only slightly worse than a profile model with one post hoc residual covariance; the profiles should therefore be regarded as an exploratory typology rather than discrete types.

Fourth, the questionnaire had no “not applicable” option, so an activity outside a respondent’s role may have been rated as low practice, although restricting the analysis to clinical staff did not change the solution. Fifth, the sample size limited precision. Simulations indicated that a three-profile structure of the observed form can be recovered with N = 213 when spurious solutions are excluded. However, power to detect the observed age association was modest (62.5% of all simulated samples and 66.8% of those in which the full procedure could be completed), and bootstrap intervals that incorporated profile-estimation uncertainty were wide and included 1. The associations with age and occupation should therefore be regarded as exploratory. Sixth, age and length of employment were highly correlated, and there was no variation in patient-facing experience, so associated factors could not be fully evaluated. Seventh, this study did not target nurses exclusively, and nurses and nursing assistants were combined in the main analysis. In addition, because the overall association of occupation was not significant, the latent profiles derived in this study cannot be interpreted as privacy protection practice types unique to nurses. Therefore, the nursing implications of this study are a practical interpretation based on the close link between the measured behaviors and routine information management tasks in nursing, and future studies need to directly verify the reproducibility of the profiles and the associated factors in multicenter samples of nurses.

## 5. Conclusions

The LPA using 11 self-reported practice items retained through Rasch-based screening identified three profiles: the Low-practice profile, the Moderate-practice profile, and the High-practice profile. In total, 54.5% of the participants reported consistently high practice, and 45.5% reported lower practice on some or all items. In particular, providing only the minimum necessary information during consultation and appropriately disposing of records past their retention period were relatively low across multiple profiles. These results are consistent with the view that patient privacy protection should be approached not as the sole responsibility of the information security department but as a patient safety and quality management issue in the clinical settings where patient information is generated and exchanged. In clinical practice, it may be important to translate privacy protection principles into concrete behaviors during handoffs, patient and caregiver communication, interdisciplinary collaboration, EMR use, and record management.

Rather than defining at-risk groups by age or occupation, healthcare institutions may consider tailoring education to the behaviors with lower practice and designing information-sharing, authentication, and record management procedures that are easy to comply with. In particular, strategies may be considered that link privacy protection to patient advocacy, professional ethics, patient safety, and informatics competencies in interprofessional education for newly hired and current staff and that provide repeated training centered on actual clinical cases. Future studies need to confirm the reproducibility of the profiles in multicenter samples of healthcare workers, distinguish between nurses and nursing assistants, and test whether profile-specific education and system improvements change practice in longitudinal and interventional studies.

## Figures and Tables

**Table 1:** Measurement indicators used in the latent profile analysis.

Item	Domain	Content (Higher Score = Better Practice)	Scoring
Item 1	Privacy	Not viewing records of other departments unrelated to one’s duties	Reverse-coded
Item 2	Privacy	Not discussing patient conditions with unrelated staff	Reverse-coded
Item 3	Privacy	Protecting sensitive conversations in multi-bed rooms and using a private space when needed	—
Item 4	Privacy	Providing only the minimum information necessary during consultation or referral	Reverse-coded
Item 5	Privacy	Not disclosing a patient’s admission or treatment status to acquaintances without authorization	Reverse-coded
Item 6	Security	Changing medical information system passwords periodically	—
Item 7	Security	Logging out when finishing system use or leaving the workstation	—
Item 8	Privacy	Not sharing medical information system IDs/passwords with colleagues	Reverse-coded
Item 9	Security	Storing paper charts, photographs, and other records in lockable locations	—
Item 10	Security	Disposing of work-related patient notes immediately after the task is completed	—
Item 11	Security	Disposing of records past their retention period or requesting disposal from the responsible department	—

Note: Reverse-coded items (Items 1, 2, 4, 5, and 8) asked about inappropriate behavior and were recoded so that higher scores consistently indicate better patient privacy protection practice. Items marked with a dash (—) were positively worded and retain their original scoring direction; thus, Item 3 is the only positively worded Privacy item, and all Security items are positively worded.

**Table 2:** General characteristics by latent profile (N = 213).

Characteristic	Category	n	Low-Practice, n (%)	Moderate-Practice, n (%)	High-Practice, n (%)
Occupation	Physician	20	1 (5.0)	6 (30.0)	13 (65.0)
	Nursing staff	115	24 (20.9)	36 (31.3)	55 (47.8)
	Administrative/support	43	8 (18.6)	10 (23.3)	25 (58.1)
	Other healthcare professionals	35	2 (5.7)	10 (28.6)	23 (65.7)
Age	<40 years	81	12 (14.8)	16 (19.8)	53 (65.4)
	40–49 years	50	14 (28.0)	13 (26.0)	23 (46.0)
	≥50 years	82	9 (11.0)	33 (40.2)	40 (48.8)
Length of employment	<5 years	71	11 (15.5)	19 (26.8)	41 (57.7)
	5–<20 years	75	17 (22.7)	15 (20.0)	43 (57.3)
	≥20 years	67	7 (10.4)	28 (41.8)	32 (47.8)

Note: Percentages are row percentages. Profile membership was assigned by maximum posterior probability; associations with profile membership were tested with the classification error-corrected three-step model (Section 3.5).

**Table 3:** Fit indices for each latent profile model.

K	Log-Likelihood	AIC	BIC	SA-BIC	Entropy	Smallest Class	AvePP Range	Status
1	−3075.31	6194.62	6268.57	6198.86	–	213 (100.0%)	–	admissible
2	−2800.52	5669.05	5783.33	5675.59	0.900	88 (41.3%)	0.974–0.975	admissible
3	−2743.24	5578.48	5733.10	5587.34	0.915	35 (16.4%)	0.955–0.971	admissible (selected)
4	−2691.71	5499.41	5694.37	5510.58	0.927	2 (0.9%)	0.940–1.000	excluded: very small class
5	−2639.14	5418.28	5653.57	5431.76	0.947	5 (2.3%)	0.927–1.000	excluded: very small class

Note: Although the information criteria decreased in the 4- and 5-profile models, these models were not adopted as the final model because their smallest groups accounted for only 0.9% and 2.3% of the total sample, respectively. The best log-likelihood was replicated in 400 additional random starts for K = 2 and 3; for K = 4 and 5, solutions with higher log-likelihoods were spurious (pooled within-profile variance of one indicator below the 0.10 criterion: 0.02 and 0.004, respectively) and are not shown (Supplementary Table S2).

**Table 4:** Estimated item means for the final three-profile model.

Item	Domain	Low-Practice	Moderate-Practice	High-Practice
Item 1	Privacy	3.48	4.20	4.72
Item 2	Privacy	2.71	4.15	4.85
Item 3	Privacy	3.77	3.92	4.72
Item 4	Privacy	2.67	3.35	3.97
Item 5	Privacy	3.65	4.02	4.71
Item 6	Security	3.91	3.73	4.76
Item 7	Security	3.65	3.75	4.81
Item 8	Privacy	3.08	3.70	4.45
Item 9	Security	3.51	3.93	4.73
Item 10	Security	3.51	3.84	4.58
Item 11	Security	3.31	3.26	4.24
-	**Privacy mean**	**3.23**	**3.89**	**4.57**
-	**Security mean**	**3.58**	**3.70**	**4.62**

Note: Scores range from 1 to 5, and higher scores indicate better practice. Five of the six Privacy items (Items 1, 2, 4, 5, and 8) were reverse-worded items about inappropriate behavior, whereas Item 3 and all Security items were positively worded; differences between the Privacy and Security means therefore coincide with differences in item wording. Domain means are shown in bold.

**Table 5:** Multinomial logistic regression of latent profile membership corrected for classification error (3-step approach).

Profile (vs. High-Practice)	Predictor	OR	95% CI	*p*
Low-practice	Age 40–49	2.26	0.86–5.95	0.098
	Age ≥ 50	0.69	0.25–1.95	0.486
	Physician	0.17	0.02–1.67	0.129
	Administrative/support	0.65	0.24–1.74	0.393
	Other healthcare professionals	0.17	0.03–0.87	0.033
Moderate-practice	Age 40–49	2.00	0.75–5.34	0.169
	Age ≥ 50	2.89	1.25–6.70	0.013
	Physician	1.04	0.31–3.49	0.943
	Administrative/support	0.69	0.27–1.80	0.454
	Other healthcare professionals	0.85	0.32–2.25	0.739

Note: Reference outcome: High-practice profile; reference age group: under 40 years; reference occupation: nursing staff. Overall *p*-value for the age variable block, *p* = 0.008; overall *p*-value for the occupation variable block, *p* = 0.141. OR, odds ratio; CI, Wald confidence interval. Bootstrap percentile intervals that incorporate profile-estimation uncertainty are given in Supplementary Table S6; for age ≥ 50 (Moderate-practice vs. High-practice), this interval included 1 (0.42–14.39).

## Data Availability

The data presented in this study are available on request from the corresponding author due to privacy restrictions. The analysis code is provided as Supplementary File S1.

## Supplementary Materials

The following supporting information can be downloaded at https://www.mdpi.com/article/10.3390/healthcare14213623/s1, Table S1: Rasch-based screening of the practice items; Table S2: Model enumeration diagnostics and bootstrap likelihood ratio tests; Table S3: Alternative model specifications; Table S4: Resampling stability and Monte Carlo simulations; Table S5: Sensitivity analyses; Table S6: Employment-duration model and bootstrap intervals for the main regression model; Table S7: Classification error matrix, estimation settings, and validation; File S1: Python code for the latent profile analysis. References [22,23,24,27,28,30,32,33,34,36] are cited in the Supplementary Materials.

## Abbreviations

EMR	Electronic Medical Record
HIS	Hospital Information System
LPA	Latent Profile Analysis
EM	Expectation-Maximization
AIC	Akaike Information Criterion
BIC	Bayesian Information Criterion
SA-BIC	Sample-size Adjusted Bayesian Information Criterion
AvePP	Average Posterior Probability
BFGS	Broyden–Fletcher–Goldfarb–Shanno
ORs	Odds Ratios
ARI	Adjusted Rand Index
BLRT	Bootstrap Likelihood Ratio Test
CI	Confidence Interval
LR	Likelihood Ratio
