# Supplementary material for: Latent Profiles of Self-Reported Patient Privacy Protection Practices Among Hospital Healthcare Workers: Implications for Patient Safety and Interprofessional Education
Source: Healthcare (Basel). 2026 Oct 26;14(21):3623. doi: 10.3390/healthcare14213623 (PMC13762769; doi:10.3390/healthcare14213623)
Supplement: Supplementary file 1 [file healthcare-14-03623-s001.zip › File_S1_analysis_code.pdf]

Supplementary File S1. Python code for the latent profile analysis

Article: Latent Profiles of Self-Reported Patient Privacy Protection Practices among Hospital Healthcare Workers: Implications for Patient Safety and Interprofessional Education (*Healthcare*).

This notebook reproduces every result in the manuscript and in Supplementary Tables S1–S7 from the item-level survey data. Run the cells from top to bottom ("Run All"); the saved outputs come from a single uninterrupted run. A PDF copy of this notebook with all outputs is provided for readers without Jupyter.

- **Part A** reproduces the original analyses (Tables 2–5, Figure 1).
- **Part B** reproduces the analyses added during peer review (Tables S1–S7).

Admissibility criterion. A latent profile solution is classified as spurious (inadmissible) when the pooled within-profile variance of any indicator is below 0.10 (`ADMISSIBLE_MIN_VAR`). The criterion is applied in the model enumeration, the bootstrap likelihood ratio tests, the nonparametric bootstrap, the Monte Carlo simulations and the sensitivity analyses: in each fit, the admissible solution with the largest log-likelihood across the starting values is retained, and fits without an admissible solution are counted and excluded. These counts, the convergence of all starts and the number of usable replications are printed in Part B. In the second revision, this criterion was added to the bootstrap and simulation procedures, which had previously retained the highest-likelihood solution without screening.

Data. The survey file (`설문지 취합 결과*.xlsx`, sheet `취합결과`) is available from the author on reasonable request. Place it in the same folder as this notebook.

Runtime. Part A takes less than a minute. Part B takes about 30–40 minutes on a standard computer, mostly for the bootstrap likelihood ratio tests, the bootstrap resampling and the Monte Carlo simulations. All random seeds are fixed, so the printed values match the manuscript and the Supplementary Tables.

Software. The original analyses (Part A) were run with Python 3.14.6, NumPy, pandas, SciPy, statsmodels and Matplotlib. The outputs saved in this file were produced with Python 3.13.16, NumPy 2.5.3, SciPy 1.18.1, pandas 3.0.5, scikit-learn 1.9.1 and Matplotlib 3.11.2; when statsmodels is not installed, an equivalent central-difference Hessian is used.

Part A. Original analyses

In [1]:

```
from pathlib import Path
import warnings
warnings.filterwarnings('ignore')

import numpy as np
import pandas as pd
import matplotlib
import matplotlib.pyplot as plt
from scipy.special import logsumexp
from scipy.optimize import minimize, linear_sum_assignment
from scipy.stats import chi2, chi2_contingency, norm, spearmanr
from sklearn.metrics import adjusted_rand_score as ARI
from sklearn.decomposition import FactorAnalysis
from sklearn.mixture import GaussianMixture
from scipy.stats import multivariate_normal

ADMISSIBLE_MIN_VAR = 0.10   # a solution is spurious if any pooled within-profile variance is
```

```
below this value

try:
    from statsmodels.tools.numdiff import approx_hess   # used in the original analysis
except ImportError:
    def approx_hess(x, f, epsilon=1e-5):                  # equivalent central-difference Hessian
        x = np.asarray(x, float); k = len(x); H = np.zeros((k, k)); h = np.full(k, epsilon)
        for i in range(k):
            for j in range(i, k):
                ei = np.zeros(k); ej = np.zeros(k); ei[i] = h[i]; ej[j] = h[j]
                H[i, j] = H[j, i] = (f(x + ei + ej) - f(x + ei - ej) - f(x - ei + ej) + f(x - ei
- ej)) / (4 * h[i] * h[j])
        return H

import sys, scipy, sklearn
print('Python', sys.version.split()[0], '| NumPy', np.__version__, '| pandas', pd.__version__,
'| SciPy', scipy.__version__,
      '| scikit-learn', sklearn.__version__, '| Matplotlib', matplotlib.__version__,
      '| statsmodels', 'installed' if 'statsmodels' in sys.modules else 'not installed (central-
difference Hessian used)')
```

```
Python 3.13.16 | NumPy 2.5.3 | pandas 3.0.5 | SciPy 1.18.1 | scikit-learn 1.9.1 | Matplotlib
3.11.2 | statsmodels not installed (central-difference Hessian used)
```

In [2]:

```
# A1. Data: the survey file must be in the notebook folder
BASE = Path.cwd()
candidates = sorted(BASE.glob('설문지 취합 결과*.xlsx'))
if not candidates:
    raise FileNotFoundError(f'Survey data file not found in {BASE}')
RAW = candidates[0]
df = pd.read_excel(RAW, sheet_name='취합결과')
print('Data file:', RAW.name, '| shape:', df.shape)
assert len(df) == 213

# The 11 practice indicators (columns of the competency diagnosis questionnaire)
vars11 = ['PIP_15', 'PIP_21', 'PIP_23', 'PIP_25', 'PIP_27',
          'PIP_32', 'PIP_34', 'PIP_36', 'PIP_38', 'PIP_43', 'PIP_45']
assert all(c in df.columns for c in vars11)
assert df[vars11].notna().all().all(), 'missing values in the indicators'
```

```
Data file: 설문지 취합 결과.xlsx | shape: (213, 52)
```

In [3]:

```
# A2. Indicator coding: higher score = better self-reported practice
item_info = pd.DataFrame({
    'Item': [f'Item {i}' for i in range(1, 12)],
    'Raw': vars11,
    'Domain': ['Privacy', 'Privacy', 'Privacy', 'Privacy', 'Privacy',
               'Security', 'Security', 'Privacy', 'Security', 'Security', 'Security'],
    'Reverse': [True, True, False, True, True, False, False, True, False, False, False]})
Xdf = df[vars11].astype(float).copy()
for c in item_info.loc[item_info['Reverse'], 'Raw']:
    Xdf[c] = 6 - Xdf[c]
Xdf.columns = item_info['Item']
X = Xdf.to_numpy(float)
raw11 = df[vars11].astype(float).to_numpy()
```

```
assert X.shape == (213, 11) and np.isin(X, [1, 2, 3, 4, 5]).all()
print(item_info)
```

```
       Item     Raw    Domain  Reverse
0    Item 1  PIP_15   Privacy     True
1    Item 2  PIP_21   Privacy     True
2    Item 3  PIP_23   Privacy    False
3    Item 4  PIP_25   Privacy     True
4    Item 5  PIP_27   Privacy     True
5    Item 6  PIP_32  Security    False
6    Item 7  PIP_34  Security    False
7    Item 8  PIP_36   Privacy     True
8    Item 9  PIP_38  Security    False
9   Item 10  PIP_43  Security    False
10  Item 11  PIP_45  Security    False
```

In [4]:

```
# A3. Covariates used in the analysis
age_code = df['2.연령'].astype(int).to_numpy()        # age category
tenure_code = df['3.근무기간'].astype(int).to_numpy()  # length of employment
occ_code = df['1.직종'].astype(int).to_numpy()         # occupation
age_group = np.where(age_code <= 2, '<40', np.where(age_code == 3, '40-49', '>=50'))
tenure_group = np.where(tenure_code == 1, '<5', np.where(np.isin(tenure_code, [2, 3, 4]), '5-
<20', '>=20'))
occ_group = np.where(occ_code == 1, 'Physician',
            np.where(np.isin(occ_code, [3, 4]), 'Nursing',
            np.where(np.isin(occ_code, [2, 9]), 'Admin/support', 'Other health')))
print(pd.Series(age_group).value_counts().to_dict())
print(pd.Series(tenure_group).value_counts().to_dict())
print(pd.Series(occ_group).value_counts().to_dict())
assert pd.Series(age_group).value_counts().to_dict() == {'>=50': 82, '<40': 81, '40-49': 50}
assert pd.Series(tenure_group).value_counts().to_dict() == {'5-<20': 75, '<5': 71, '>=20': 67}
assert pd.Series(occ_group).value_counts().to_dict() == {'Nursing': 115, 'Admin/support': 43,
'Other health': 35, 'Physician': 20}

# Spearman correlation between the ordinal age and employment categories
age_num = np.where(age_group == '<40', 1, np.where(age_group == '40-49', 2, 3))
ten_num = np.where(tenure_group == '<5', 1, np.where(tenure_group == '5-<20', 2, 3))
rho, p_rho = spearmanr(age_num, ten_num)
print(f"Spearman rho = {rho:.3f}, p = {p_rho:.2e}")
assert round(rho, 3) == .786
```

```
{'>=50': 82, '<40': 81, '40-49': 50}
{'5-<20': 75, '<5': 71, '>=20': 67}
{'Nursing': 115, 'Admin/support': 43, 'Other health': 35, 'Physician': 20}
Spearman rho = 0.786, p = 5.58e-46
```

In [5]:

```
# A4. Gaussian latent profile model (equal variances across profiles, diagonal covariance), EM
estimation
def fit_lpa_shared_diag(X, K, n_starts=50, seed=20260907, max_iter=3000, tol=1e-9):
    rng = np.random.default_rng(seed)
    n, d = X.shape
    global_var = np.maximum(np.var(X, axis=0, ddof=0), 1e-8)
    starts = []
    if K == 1:
        starts = [(np.array([1.0]), X.mean(0, keepdims=True), global_var.copy())]
    else:
```

```
        score = X.mean(1)
        qs = np.quantile(score, np.linspace(0, 1, K + 2)[1:-1])
        lab0 = np.digitize(score, qs)
        means0 = np.vstack([X[lab0 == k].mean(0) if np.any(lab0 == k) else X[rng.integers(n)]
for k in range(K)])
        pi0 = np.array([(lab0 == k).mean() for k in range(K)]); pi0 = np.maximum(pi0, 1e-6); pi0
/= pi0.sum()
        starts.append((pi0, means0, global_var.copy()))
        for _ in range(n_starts - 1):
            idx = rng.choice(n, K, replace=False)
            starts.append((rng.dirichlet(np.ones(K)), X[idx].copy() + rng.normal(0, 0.05, size=
(K, d)), global_var.copy()))
    best = None
    for pi, means, var in starts:
        prev_ll = -np.inf
        for _ in range(max_iter):
            logdens = -0.5 * (np.sum(np.log(2 * np.pi * var)) + np.sum((X[:, None, :] -
means[None, :, :]) ** 2 / var[None, None, :], axis=2))
            logjoint = logdens + np.log(np.maximum(pi, 1e-300))[None, :]
            lse = logsumexp(logjoint, axis=1); ll = lse.sum(); resp = np.exp(logjoint - lse[:,
None])
            Nk = resp.sum(0); pi = Nk / n; means = (resp.T @ X) / Nk[:, None]
            var = np.maximum((resp[:, :, None] * (X[:, None, :] - means[None, :, :]) **
2).sum(axis=(0, 1)) / n, 1e-8)
            if abs(ll - prev_ll) < tol * (1 + abs(ll)):
                break
            prev_ll = ll
        logdens = -0.5 * (np.sum(np.log(2 * np.pi * var)) + np.sum((X[:, None, :] - means[None,
:, :]) ** 2 / var[None, None, :], axis=2))
        logjoint = logdens + np.log(np.maximum(pi, 1e-300))[None, :]
        lse = logsumexp(logjoint, axis=1); ll = lse.sum(); resp = np.exp(logjoint - lse[:,
None])
        if best is None or ll > best['ll']:
            best = {'ll': ll, 'pi': pi.copy(), 'means': means.copy(), 'var': var.copy(), 'resp':
resp.copy()}
    p = K * d + d + (K - 1)
    best.update(parameters=p, aic=-2 * best['ll'] + 2 * p, bic=-2 * best['ll'] + p * np.log(n),
                sabic=-2 * best['ll'] + p * np.log((n + 2) / 24))
    hard = np.argmax(best['resp'], axis=1); best['hard'] = hard; best['counts'] =
np.bincount(hard, minlength=K)
    r = best['resp']
    best['entropy'] = np.nan if K == 1 else 1 + np.sum(r * np.log(np.maximum(r, 1e-300))) / (n *
np.log(K))
    best['avepp'] = np.array([1.0]) if K == 1 else np.array([r[hard == k, k].mean() for k in
range(K)])
    return best

fits = {K: fit_lpa_shared_diag(X, K, n_starts=1 if K == 1 else (50 if K <= 3 else 80), seed=1234
+ K) for K in range(1, 6)}
fit_table = pd.DataFrame([{'K': K, 'LL': f['ll'], 'AIC': f['aic'], 'BIC': f['bic'], 'SA-BIC':
f['sabic'],
                           'Entropy': f['entropy'], 'Smallest': int(f['counts'].min()),
                           'AvePP min': f['avepp'].min(), 'AvePP max': f['avepp'].max(),
                           'Min var': f['var'].min(), 'Admissible': bool(f['var'].min() >=
ADMISSIBLE_MIN_VAR)} for K, f in fits.items()])
print(fit_table.round(3).to_string(index=False))
print('Note: rows K = 4 and 5 here come from 80 starts; Table 3 reports the best admissible
solutions found',
      'with 400 additional random starts (Part B, Table S2).')
```

```
 K        LL      AIC      BIC   SA-BIC  Entropy  Smallest  AvePP min  AvePP max  Min var
Admissible
 1 -3075.311 6194.623 6268.571 6198.859      NaN       213      1.000      1.000    0.619
True
 2 -2800.523 5669.046 5783.330 5675.594    0.900        88      0.974      0.975    0.374
True
 3 -2743.239 5578.478 5733.098 5587.337    0.915        35      0.955      0.971    0.161
True
 4 -2691.705 5499.410 5694.365 5510.580    0.927         2      0.940      1.000    0.162
True
 5 -2639.141 5418.281 5653.572 5431.762    0.947         5      0.927      1.000    0.199
True
Note: rows K = 4 and 5 here come from 80 starts; Table 3 reports the best admissible solutions
found with 400 additional random starts (Part B, Table S2).
```

In [6]:

```
# A5. Selected three-profile model: profiles ordered by mean item score
labels = np.array(['Low-practice', 'Moderate-practice', 'High-practice'])
f3 = fits[3]
order = np.argsort(f3['means'].mean(axis=1))
R = f3['resp'][:, order]; means3 = f3['means'][order]; hard3 = np.argmax(R, axis=1)
counts3 = np.bincount(hard3, minlength=3)
avepp3 = np.array([R[hard3 == k, k].mean() for k in range(3)])
entropy3 = 1 + np.sum(R * np.log(np.maximum(R, 1e-300))) / (len(X) * np.log(3))
print('Profile sizes:', dict(zip(labels, counts3)))
print(f"Entropy = {entropy3:.3f}; AvePP = {np.round(avepp3, 3)}")
print(f"LL = {f3['ll']:.2f}, AIC = {f3['aic']:.2f}, BIC = {f3['bic']:.2f}, SA-BIC =
{f3['sabic']:.2f}")
assert counts3.tolist() == [35, 62, 116] and round(entropy3, 3) == .915 and round(f3['bic'], 2)
== 5733.10
```

```
Profile sizes: {np.str_('Low-practice'): np.int64(35), np.str_('Moderate-practice'):
np.int64(62), np.str_('High-practice'): np.int64(116)}
Entropy = 0.915; AvePP = [0.971 0.955 0.969]
LL = -2743.24, AIC = 5578.48, BIC = 5733.10, SA-BIC = 5587.34
```

In [7]:

```
# A6. Estimated item means by profile (Table 4)
profile_means = pd.DataFrame(means3.T, index=item_info['Item'], columns=labels)
profile_means.insert(0, 'Domain', item_info['Domain'].to_numpy())
priv = np.where(item_info['Domain'] == 'Privacy')[0]; secu = np.where(item_info['Domain'] ==
'Security')[0]
profile_means.loc['Privacy mean'] = ['-'] + [means3[k, priv].mean() for k in range(3)]
profile_means.loc['Security mean'] = ['-'] + [means3[k, secu].mean() for k in range(3)]
print(profile_means.round(2).to_string())
```

```
                 Domain  Low-practice  Moderate-practice  High-practice
Item
Item 1          Privacy          3.48               4.20           4.72
Item 2          Privacy          2.71               4.15           4.85
Item 3          Privacy          3.77               3.92           4.72
Item 4          Privacy          2.67               3.35           3.97
Item 5          Privacy          3.65               4.02           4.71
Item 6         Security          3.91               3.73           4.76
Item 7         Security          3.65               3.75           4.81
Item 8          Privacy          3.08               3.70           4.45
Item 9         Security          3.51               3.93           4.73
Item 10        Security          3.51               3.84           4.58
```

```
Item 11        Security         3.31            3.26         4.24
Privacy mean          -         3.23            3.89         4.57
Security mean         -         3.58            3.70         4.62
```

In [8]:

```
# A7. Figure 1
fig, ax = plt.subplots(figsize=(9.0, 5.2))
for k, lab in enumerate(labels):
    ax.plot([f'I{i}' for i in range(1, 12)], means3[k], marker='o', linewidth=1.8, label=f'{lab}
(n={counts3[k]})')
ax.set_title('Latent profiles of self-reported patient privacy protection practice')
ax.set_xlabel('Practice indicators (Items 1–11)'); ax.set_ylabel('Estimated mean score')
ax.set_ylim(1, 5.1); ax.grid(True, alpha=.25); ax.legend(loc='lower left'); fig.tight_layout()
fig.savefig(BASE / 'Figure1.png', dpi=600, bbox_inches='tight')
plt.show()
```

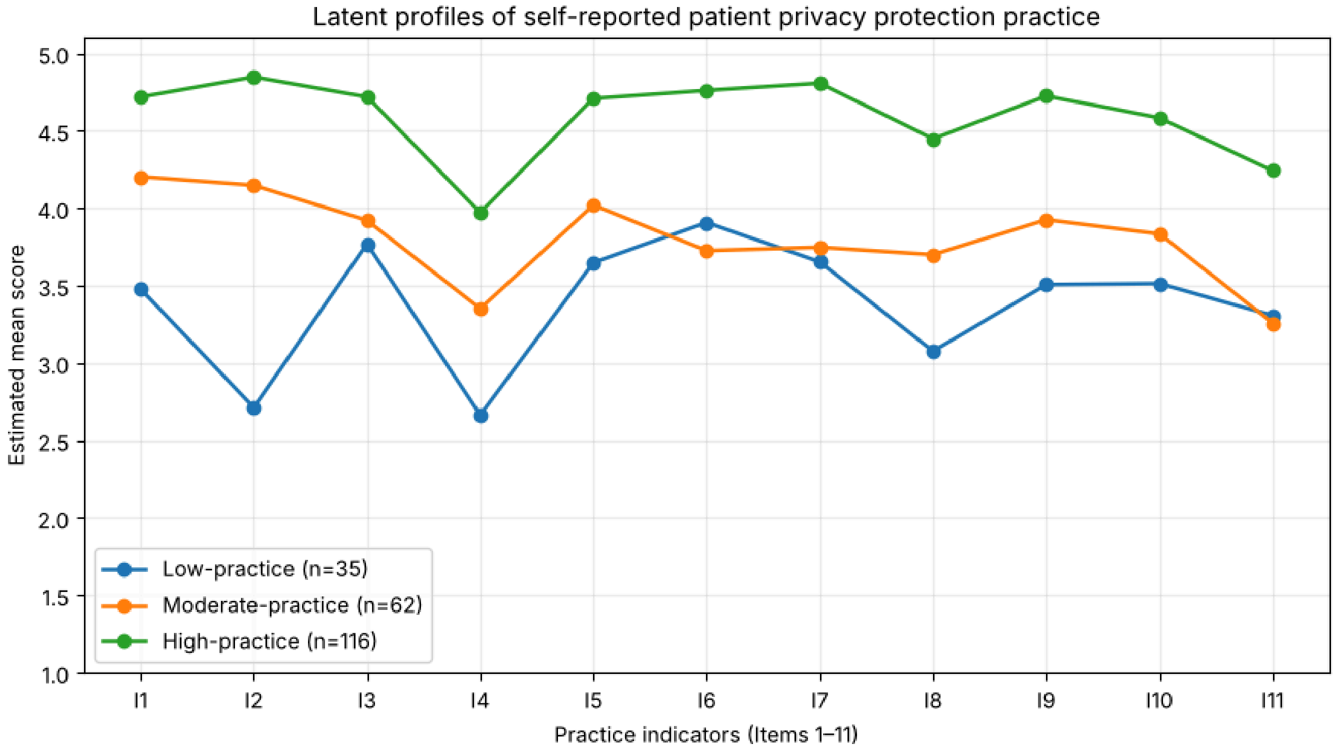

In [9]:

```
# A8. Profile membership by occupation, age and employment duration (Table 2)
for name, g, cats in [('Occupation', occ_group, ['Physician', 'Nursing', 'Admin/support', 'Other
health']),
                      ('Age', age_group, ['<40', '40-49', '>=50']),
                      ('Employment', tenure_group, ['<5', '5-<20', '>=20'])]:
    tab = pd.DataFrame([[np.sum((g == c) & (hard3 == k)) for k in range(3)] for c in cats],
index=cats, columns=labels)
    print(f'\n{name}'); print(tab)
```

Occupation

	Low-practice	Moderate-practice	High-practice
Physician	1	6	13
Nursing	24	36	55
Admin/support	8	10	25
Other health	2	10	23

```
Age
       Low-practice  Moderate-practice  High-practice
<40              12                 16             53
40-49            14                 13             23
>=50              9                 33             40

Employment
       Low-practice  Moderate-practice  High-practice
<5               11                 19             41
5-<20            17                 15             43
>=20              7                 28             32
```

In [10]:

```
# A9. Classification error-corrected three-step multinomial logistic regression (Table 5)
D = np.zeros((3, 3))                          # D[w, c] = P(assigned w | latent c)
for c in range(3):
    for w in range(3):
        D[w, c] = R[hard3 == w, c].sum() / R[:, c].sum()
print('Classification error matrix (rows assigned, columns latent):'); print(pd.DataFrame(D,
index=labels, columns=labels).round(3))

age40 = (age_group == '40-49').astype(float); age50 = (age_group == '>=50').astype(float)
phys = (occ_group == 'Physician').astype(float); admin = (occ_group ==
'Admin/support').astype(float)
other = (occ_group == 'Other health').astype(float)
Z = np.column_stack([np.ones(len(df)), age40, age50, phys, admin, other])
pred_names = ['Intercept', 'Age 40-49', 'Age >=50', 'Physician', 'Administrative/support',
'Other health']

def true_class_probs(beta_vec, Z):
    B = beta_vec.reshape(2, Z.shape[1]); eta = np.column_stack([Z @ B.T, np.zeros(len(Z))])
    return np.exp(eta - logsumexp(eta, axis=1)[:, None])

def negll_3step(beta_vec, Z, assigned, D):
    return -np.log(np.maximum((D[assigned, :] * true_class_probs(beta_vec, Z)).sum(axis=1), 1e-
300)).sum()

def fit_3step(Z, assigned, D):
    opt = minimize(negll_3step, np.zeros(2 * Z.shape[1]), args=(Z, assigned, D), method='BFGS',
options={'maxiter': 10000, 'gtol': 1e-9})
    H = approx_hess(opt.x, lambda b: -negll_3step(b, Z, assigned, D), epsilon=1e-5)
    return {'beta': opt.x, 'se': np.sqrt(np.diag(-np.linalg.inv(H))), 'll': -opt.fun}

full = fit_3step(Z, hard3, D)
B = full['beta'].reshape(2, -1); SE = full['se'].reshape(2, -1)
reg = pd.DataFrame([{'Outcome vs High': lab, 'Predictor': pred_names[j], 'OR': np.exp(B[c, j]),
                     'CI low': np.exp(B[c, j] - 1.96 * SE[c, j]), 'CI high': np.exp(B[c, j] +
1.96 * SE[c, j]),
                     'p': 2 * norm.sf(abs(B[c, j] / SE[c, j]))}
                    for c, lab in enumerate(['Low-practice', 'Moderate-practice']) for j in
range(1, 6)])
print(reg.round({'OR': 2, 'CI low': 2, 'CI high': 2, 'p': 3}).to_string(index=False))

null = fit_3step(np.ones((len(df), 1)), hard3, D)
no_age = fit_3step(np.column_stack([np.ones(len(df)), phys, admin, other]), hard3, D)
no_occ = fit_3step(np.column_stack([np.ones(len(df)), age40, age50]), hard3, D)
LR_all = 2 * (full['ll'] - null['ll']); LR_age = 2 * (full['ll'] - no_age['ll']); LR_occ = 2 *
(full['ll'] - no_occ['ll'])
print(f"Overall LR = {LR_all:.2f}, df = 10, p = {chi2.sf(LR_all, 10):.3f}; McFadden R2 = {1 -
```

```
full['ll'] / null['ll']:.3f}")
print(f"Age block p = {chi2.sf(LR_age, 4):.3f}; occupation block p = {chi2.sf(LR_occ, 6):.3f}")
```

```
Classification error matrix (rows assigned, columns latent):
                   Low-practice  Moderate-practice  High-practice
Low-practice              0.994              0.016          0.000
Moderate-practice         0.006              0.927          0.023
High-practice             0.000              0.057          0.977
  Outcome vs High              Predictor   OR  CI low  CI high     p
     Low-practice              Age 40-49 2.26    0.86     5.95 0.098
     Low-practice                Age >=50 0.69    0.25     1.95 0.486
     Low-practice              Physician 0.17    0.02     1.67 0.129
     Low-practice Administrative/support 0.65    0.24     1.74 0.393
     Low-practice           Other health 0.17    0.03     0.87 0.033
Moderate-practice              Age 40-49 2.00    0.75     5.34 0.169
Moderate-practice               Age >=50 2.89    1.25     6.70 0.013
Moderate-practice              Physician 1.04    0.31     3.49 0.943
Moderate-practice Administrative/support 0.69    0.27     1.80 0.454
Moderate-practice           Other health 0.85    0.32     2.25 0.739
Overall LR = 23.72, df = 10, p = 0.008; McFadden R2 = 0.056
Age block p = 0.008; occupation block p = 0.141
```

In [11]:

```
# A10. Sensitivity model: employment duration in place of age (block test; complete model in Part B)
ten520 = (tenure_group == '5-<20').astype(float); ten20 = (tenure_group == '>=20').astype(float)
full_ten = fit_3step(np.column_stack([np.ones(len(df)), ten520, ten20, phys, admin, other]), hard3, D)
no_ten = fit_3step(np.column_stack([np.ones(len(df)), phys, admin, other]), hard3, D)
LR_ten = 2 * (full_ten['ll'] - no_ten['ll'])
print(f"Employment-duration block: LR = {LR_ten:.2f}, df = 4, p = {chi2.sf(LR_ten, 4):.3f}")
```

```
Employment-duration block: LR = 10.93, df = 4, p = 0.027
```

Part B. Analyses added during peer review (Supplementary Tables S1–S7)

In [12]:

```
# B0. Helper functions used in Part B
def lpa_fit(X, K, n_starts=50, seed=0, shared=True, max_iter=3000, tol=1e-9, var_floor=1e-3, min_var=None):
    # Gaussian mixture with diagonal covariance; shared=True: variances equal across profiles.
    # min_var: return the admissible solution (every within-profile variance >= min_var) with the largest
    # log-likelihood across starts; 'admissible' is False when no start gave one (the best solution is then
    # returned and must not be used). 'replaced' is True when the highest-likelihood solution was inadmissible.
    # 'diag' counts how the starts ended: converged, stopped because a profile emptied, or reached max_iter.
    rng = np.random.default_rng(seed); n, d = X.shape; gv = np.var(X, axis=0)
    def logjoint(pi, means, var):
        if shared:
            ld = -0.5 * (np.sum(np.log(2 * np.pi * var)) + np.sum((X[:, None, :] - means[None]) ** 2 / var[None, None, :], axis=2))
        else:
            ld = -0.5 * (np.sum(np.log(2 * np.pi * var), axis=1)[None, :] + np.sum((X[:, None, :] - means[None]) ** 2 / var[None], axis=2))
        return ld + np.log(np.maximum(pi, 1e-300))[None, :]
```

```
    if K == 1:
        starts = [(np.ones(1), X.mean(0, keepdims=True))]
    else:
        score = X.mean(1); qs = np.quantile(score, np.linspace(0, 1, K + 2)[1:-1]); lab =
np.digitize(score, qs)
        m0 = np.vstack([X[lab == k].mean(0) if np.any(lab == k) else X[rng.integers(n)] for k in
range(K)])
        p0 = np.maximum(np.array([(lab == k).mean() for k in range(K)]), 1e-6); p0 /= p0.sum()
        starts = [(p0, m0)]
        for _ in range(n_starts - 1):
            idx = rng.choice(n, K, replace=False)
            starts.append((rng.dirichlet(np.ones(K)), X[idx] + rng.normal(0, .05, (K, d))))
    best = None; best_adm = None; diag = {'converged': 0, 'empty': 0, 'max_iter': 0}
    for pi, means in starts:
        var = gv.copy() if shared else np.tile(gv, (K, 1)); prev = -np.inf; status = 'max_iter'
        for _ in range(max_iter):
            lj = logjoint(pi, means, var); lse = logsumexp(lj, axis=1); ll = lse.sum(); r =
np.exp(lj - lse[:, None]); Nk = r.sum(0)
            if np.any(Nk < 1e-8):
                status = 'empty'; break
            pi = Nk / n; means = (r.T @ X) / Nk[:, None]; sq = (X[:, None, :] - means[None]) **
2
            var = np.maximum((r[:, :, None] * sq).sum((0, 1)) / n, var_floor) if shared else
np.maximum((r[:, :, None] * sq).sum(0) / Nk[:, None], var_floor)
            if abs(ll - prev) < tol * (1 + abs(ll)):
                status = 'converged'; break
            prev = ll
        diag[status] += 1
        lj = logjoint(pi, means, var); lse = logsumexp(lj, axis=1); ll = lse.sum(); r =
np.exp(lj - lse[:, None])
        if not np.isfinite(ll):
            continue
        sol = dict(ll=ll, pi=pi.copy(), means=means.copy(), var=var.copy(), resp=r.copy())
        if best is None or ll > best['ll']:
            best = sol
        if min_var is not None and status != 'empty' and var.min() >= min_var and (best_adm is
None or ll > best_adm['ll']):
            best_adm = sol
    if min_var is None:
        out = best; out['admissible'] = None; out['replaced'] = False
    elif best_adm is not None:
        out = best_adm; out['admissible'] = True; out['replaced'] = best_adm is not best
    else:
        out = best; out['admissible'] = False; out['replaced'] = False
    out['diag'] = diag; out['ll_any'] = best['ll']
    p = K * d + (d if shared else K * d) + (K - 1)
    out.update(p=p, aic=-2 * out['ll'] + 2 * p, bic=-2 * out['ll'] + p * np.log(n), sabic=-2 *
out['ll'] + p * np.log((n + 2) / 24))
    h = out['resp'].argmax(1); out['hard'] = h; out['counts'] = np.bincount(h, minlength=K); r =
out['resp']
    out['entropy'] = np.nan if K == 1 else 1 + np.sum(r * np.log(np.maximum(r, 1e-300))) / (n *
np.log(K))
    return out

def lpa_simulate(f, n, rng):
    K = len(f['pi']); z = rng.choice(K, n, p=f['pi'])
    return f['means'][z] + rng.normal(size=(n, f['means'].shape[1])) * np.sqrt(f['var'])

def hess_num(x, f, step=1e-4):
    x = np.asarray(x, float); k = len(x); H = np.zeros((k, k)); h = step * np.maximum(np.abs(x),
```

```
1.0)
    for i in range(k):
        for j in range(i, k):
            ei = np.zeros(k); ej = np.zeros(k); ei[i] = h[i]; ej[j] = h[j]
            H[i, j] = H[j, i] = (f(x + ei + ej) - f(x + ei - ej) - f(x - ei + ej) + f(x - ei -
ej)) / (4 * h[i] * h[j])
    return H

def error_matrix(R, h):
    D = np.zeros((3, 3))
    for c in range(3):
        for w in range(3):
            D[w, c] = R[h == w, c].sum() / max(R[:, c].sum(), 1e-12)
    return D

def three_step(Zd, h, D, se=True, gtol=1e-9, maxiter=10000, absval=True):
    def nll(b):
        Bm = b.reshape(2, Zd.shape[1]); eta = np.column_stack([Zd @ Bm.T, np.zeros(len(Zd))])
        p = np.exp(eta - logsumexp(eta, 1)[:, None]); return -np.log(np.maximum((D[h, :] *
p).sum(1), 1e-300)).sum()
    op = minimize(nll, np.zeros(2 * Zd.shape[1]), method='BFGS', options={'gtol': gtol,
'maxiter': maxiter})
    s = None
    if se:
        try:
            v = np.diag(-np.linalg.inv(hess_num(op.x, lambda b: -nll(b))))
        except np.linalg.LinAlgError:                       # singular Hessian: standard errors
not estimable
            v = np.full(op.x.size, np.nan)
        with np.errstate(invalid='ignore'):
            s = np.sqrt(np.abs(v) if absval else v)
    return op.x.reshape(2, -1), (s.reshape(2, -1) if se else None), -op.fun

def ordered_fit(Xd, starts, seed, min_var=ADMISSIBLE_MIN_VAR):
    # three-profile model; profiles ordered by mean item score (Low, Moderate, High), as in the
main analysis
    f = lpa_fit(Xd, 3, n_starts=starts, seed=seed, min_var=min_var); o =
np.argsort(f['means'].mean(1))
    R_ = f['resp'][:, o]; h_ = R_.argmax(1); return f, o, R_, h_, error_matrix(R_, h_)

def matches_reference(M_ordered, M_ref_ordered):
    # True when ordering by mean item score gives the same labels as Hungarian matching on
profile means
    _, cols = linear_sum_assignment(((M_ordered[:, None, :] - M_ref_ordered[None]) ** 2).sum(2))
    return bool(np.all(cols == np.arange(len(cols)))), cols

def add_diag(total, g):
    for k_ in total:
        total[k_] += g['diag'][k_]
    return total

n = len(X); h_main = hard3.copy()
NO = df['NO'].to_numpy()
```

In [13]:

```
# B1. Rasch rating scale screening of the 18 practice items (Table S1)
P_COLS = ['PIP_9', 'PIP_11', 'PIP_13', 'PIP_15', 'PIP_19', 'PIP_21', 'PIP_23', 'PIP_25',
'PIP_27',
          'PIP_30', 'PIP_32', 'PIP_34', 'PIP_36', 'PIP_38', 'PIP_40', 'PIP_41', 'PIP_43',
```

```
'PIP_45']   # P1-P18
NEG = [1, 2, 3, 4, 6, 8, 9, 10, 13]                                                    #
negatively keyed items
X18 = df[P_COLS].astype(int).to_numpy().copy()
for i in NEG:
    X18[:, i - 1] = 6 - X18[:, i - 1]
X18 = X18 - 1                                                                          #
categories 0-4

def rsm_probs(theta, delta, tau):
    k = np.arange(len(tau) + 1); cum = np.concatenate([[0], np.cumsum(tau)])
    eta = k[None, None, :] * (theta[:, None, None] - delta[None, :, None]) - cum[None, None, :]
    eta -= eta.max(2, keepdims=True); P = np.exp(eta); return P / P.sum(2, keepdims=True)

def rsm_jmle(Xc, iters=500, tol=1e-6):
    N, L = Xc.shape; m = 4; theta = np.zeros(N); delta = np.zeros(L); tau = np.zeros(m); k =
np.arange(m + 1)
    score = Xc.sum(1); ext = (score == 0) | (score == m * L); act = ~ext
    for _ in range(iters):
        P = rsm_probs(theta, delta, tau); E = (P * k).sum(2); V = (P * k ** 2).sum(2) - E ** 2
        dth = np.zeros(N); dth[act] = (Xc - E)[act].sum(1) / np.maximum(V[act].sum(1), 1e-9);
theta += np.clip(dth, -1, 1)
        P = rsm_probs(theta, delta, tau); E = (P * k).sum(2); V = (P * k ** 2).sum(2) - E ** 2
        dd = -((Xc - E) * act[:, None]).sum(0) / np.maximum((V * act[:, None]).sum(0), 1e-9);
delta += np.clip(dd, -1, 1); delta -= delta.mean()
        P = rsm_probs(theta, delta, tau)
        for j in range(1, m + 1):
            pg = P[:, :, j:].sum(2); g = ((pg - (Xc >= j)) * act[:, None]).sum(); hh = (pg * (1
- pg) * act[:, None]).sum()
            tau[j - 1] += np.clip(g / max(hh, 1e-9), -1, 1)
        tau -= tau.mean()
        if max(np.abs(dth).max(), np.abs(dd).max()) < tol:
            break
    for i in np.where(ext)[0]:                                                         #
extreme scores: 0.3-point correction
        target = score[i] + (0.3 if score[i] == 0 else -0.3); t = theta[i]
        for _ in range(200):
            Pn = rsm_probs(np.array([t]), delta, tau)[0]; E = (Pn * k).sum(1); V = (Pn * k **
2).sum(1) - E ** 2
            t += np.clip((target - E.sum()) / max(V.sum(), 1e-9), -1, 1)
        theta[i] = t
    return theta, delta, tau, ext

def rsm_fit(Xc, theta, delta, tau, axis, use=None):
    k = np.arange(len(tau) + 1); P = rsm_probs(theta, delta, tau)
    E = (P * k).sum(2); W = (P * k ** 2).sum(2) - E ** 2; C = (P * (k[None, None, :] - E[:, :,
None]) ** 4).sum(2); Y = Xc - E
    mask = np.ones_like(Xc, bool) if use is None else np.broadcast_to(use, Xc.shape)
    cnt = mask.sum(axis); outfit = (Y ** 2 / W * mask).sum(axis) / cnt; infit = (Y ** 2 *
mask).sum(axis) / (W * mask).sum(axis)
    qo = np.sqrt(np.maximum(((C / W ** 2) * mask).sum(axis) / cnt ** 2 - 1 / cnt, 1e-12))
    qi = np.sqrt(np.maximum(((C - W ** 2) * mask).sum(axis) / ((W * mask).sum(axis)) ** 2, 1e-
12))
    return infit, (np.cbrt(infit) - 1) * (3 / qi) + qi / 3, outfit, (np.cbrt(outfit) - 1) * (3 /
qo) + qo / 3

def rsm_se(Xc, theta, delta, tau, axis, use_rows=None):
    k = np.arange(len(tau) + 1); P = rsm_probs(theta, delta, tau); E = (P * k).sum(2); W = (P *
k ** 2).sum(2) - E ** 2
    if use_rows is not None:
```

```
        W = W * use_rows[:, None]
    return 1 / np.sqrt(W.sum(axis))

def reliability(est, ses):
    ov = np.var(est, ddof=1); ev = np.mean(ses ** 2); tv = max(ov - ev, 0); return tv / ov,
np.sqrt(tv / ev)

th, de, ta, ext = rsm_jmle(X18)
p_in, p_zi, p_out, p_zo = rsm_fit(X18, th, de, ta, axis=1)
order_p = np.argsort(-np.where(ext, -np.inf, p_in)); excl11 = order_p[:11]
print('Respondents excluded for person misfit (NO):', sorted(NO[excl11].tolist()), '| infit of
11th and 12th:', np.round(p_in[order_p[10:12]], 2))
keep202 = np.setdiff1d(np.arange(n), excl11); Xk = X18[keep202]
t2, d2, a2, e2 = rsm_jmle(Xk)
ii, izi, io, izo = rsm_fit(Xk, t2, d2, a2, axis=0, use=(~e2)[:, None])
si = rsm_se(Xk, t2, d2, a2, axis=0, use_rows=(~e2).astype(float))
retained = {4: 1, 6: 2, 7: 3, 8: 4, 9: 5, 11: 6, 12: 7, 13: 8, 14: 9, 17: 10, 18: 11}
tableS1 = pd.DataFrame({'Item': [f'P{i}' for i in range(1, 19)], 'Keying': ['-' if i in NEG else
'+' for i in range(1, 19)],
                        'Measure': d2.round(2), 'SE': si.round(2), 'Infit': ii.round(2), 'Infit
Z': izi.round(1),
                        'Outfit': io.round(2), 'Outfit Z': izo.round(1),
                        'Decision': [f'Retained (Item {retained[i]})' if i in retained else
'Removed' for i in range(1, 19)]})
print(tableS1.to_string(index=False))
print('Flagged by the rule (infit outside 0.6-1.4 and |Z| > 2):', tableS1.loc[((ii > 1.4) | (ii
< .6)) & (np.abs(izi) > 2), 'Item'].tolist())

ret0 = [3, 5, 6, 7, 8, 10, 11, 12, 13, 16, 17]
Xr = Xk[:, ret0]; t3, d3, a3, e3 = rsm_jmle(Xr)
rp = reliability(t3[~e3], rsm_se(Xr, t3, d3, a3, axis=1)[~e3]); ri = reliability(d3, rsm_se(Xr,
t3, d3, a3, axis=0, use_rows=(~e3).astype(float)))
k5 = np.arange(5); P = rsm_probs(t3, d3, a3); E = (P * k5).sum(2); Wv = (P * k5 ** 2).sum(2) - E
** 2
print(f'11 retained items (n = 202): person reliability {rp[0]:.2f} (separation {rp[1]:.2f});
item reliability {ri[0]:.2f} (separation {ri[1]:.2f})')
print('Thresholds:', a3.round(2))
print('Category use (%):', np.round([100 * (Xr == c).mean() for c in k5], 1))
print('Category average measures:', np.round([(t3[:, None] - d3[None, :])[Xr == c].mean() for c
in k5], 2))
print('Category outfit:', np.round([((Xr - E) ** 2 / Wv)[Xr == c].mean() for c in k5], 2))
ii3 = rsm_fit(Xr, t3, d3, a3, axis=0, use=(~e3)[:, None])[0]; print('Item infit range:',
ii3.min().round(2), '-', ii3.max().round(2))
Xall = X18[:, ret0]; t4, d4, a4, e4 = rsm_jmle(Xall)
rp_all = reliability(t4[~e4], rsm_se(Xall, t4, d4, a4, axis=1)[~e4]); print(f'All 213
respondents: person reliability {rp_all[0]:.2f} (separation {rp_all[1]:.2f})')
```

```
Respondents excluded for person misfit (NO): [5, 42, 81, 86, 122, 158, 174, 175, 176, 177, 193] |
infit of 11th and 12th: [3.32 2.99]
Item Keying  Measure   SE  Infit  Infit Z  Outfit  Outfit Z          Decision
  P1      -     1.01 0.09   1.55      4.9    1.76       6.2           Removed
  P2      -    -0.25 0.11   1.73      5.2    1.78       4.8           Removed
  P3      -     0.40 0.09   1.37      3.2    1.39       3.2           Removed
  P4      -    -0.38 0.11   0.98     -0.1    1.00       0.0  Retained (Item 1)
  P5      +    -0.49 0.11   0.59     -4.0    0.77      -1.7           Removed
  P6      -    -0.16 0.10   0.78     -2.0    0.72      -2.3  Retained (Item 2)
  P7      +    -0.43 0.11   0.86     -1.2    0.81      -1.4  Retained (Item 3)
  P8      -     0.99 0.09   1.02      0.2    1.14       1.3  Retained (Item 4)
  P9      -    -0.25 0.11   1.00      0.0    1.04       0.4  Retained (Item 5)
 P10      -     0.01 0.10   1.44      3.5    1.34       2.5           Removed
```

```
 P11      +    -0.30 0.11   1.07      0.6    1.25       1.8  Retained (Item 6)
 P12      +    -0.23 0.11   0.84     -1.4    0.99      -0.0  Retained (Item 7)
 P13      -     0.43 0.09   1.05      0.5    0.99      -0.0  Retained (Item 8)
 P14      +    -0.24 0.11   0.63     -3.6    0.76      -2.0  Retained (Item 9)
 P15      +    -0.60 0.12   0.51     -4.9    0.50      -4.1            Removed
 P16      +    -0.27 0.11   0.53     -4.9    0.56      -3.9            Removed
 P17      +    -0.00 0.10   0.80     -1.9    0.82      -1.5 Retained (Item 10)
 P18      +     0.76 0.09   1.24      2.2    1.45       3.8 Retained (Item 11)
Flagged by the rule (infit outside 0.6-1.4 and |Z| > 2): ['P1', 'P2', 'P5', 'P10', 'P15', 'P16']
11 retained items (n = 202): person reliability 0.82 (separation 2.14); item reliability 0.96
(separation 5.03)
Thresholds: [-2.33 -0.37  0.37  2.33]
Category use (%): [ 0.5  5.6 14.7 36.5 42.7]
Category average measures: [0.97 0.62 0.71 1.63 3.63]
Category outfit: [12.55  4.27  1.3   0.5   0.62]
Item infit range: 0.63 - 1.25
All 213 respondents: person reliability 0.79 (separation 1.97)
```

In [14]:

```
# B2. Model enumeration diagnostics: 400 random single starts per model (Table S2)
def single_start(Xd, K, seed, max_iter=3000, tol=1e-9):
    rng = np.random.default_rng(seed); nn, d = Xd.shape; idx = rng.choice(nn, K, replace=False)
    means = Xd[idx] + rng.normal(0, .05, (K, d)); pi = rng.dirichlet(np.ones(K)); var =
Xd.var(0); prev = -np.inf
    for _ in range(max_iter):
        lj = -0.5 * (np.sum(np.log(2 * np.pi * var)) + np.sum((Xd[:, None, :] - means[None]) **
2 / var, 2)) + np.log(np.maximum(pi, 1e-300))
        lse = logsumexp(lj, 1); ll = lse.sum(); r = np.exp(lj - lse[:, None]); Nk = r.sum(0)
        if np.any(Nk < 1e-8):
            return None
        pi = Nk / nn; means = (r.T @ Xd) / Nk[:, None]; var = np.maximum((r[:, :, None] * (Xd[:,
None, :] - means[None]) ** 2).sum((0, 1)) / nn, 1e-8)
        if abs(ll - prev) < tol * (1 + abs(ll)):
            break
        prev = ll
    lj = -0.5 * (np.sum(np.log(2 * np.pi * var)) + np.sum((Xd[:, None, :] - means[None]) ** 2 /
var, 2)) + np.log(np.maximum(pi, 1e-300))
    lse = logsumexp(lj, 1); r = np.exp(lj - lse[:, None])
    return float(lse.sum()), float(var.min()), int(var.argmin()) + 1,
sorted(np.bincount(r.argmax(1), minlength=K).tolist())

for K in range(2, 6):
    sols = [s for s in (single_start(X, K, 10000 * K + j) for j in range(400)) if s is not None]
    uniq = {}
    for ll, mv, mi, cnt in sols:
        u = uniq.setdefault(round(ll, 1), dict(ll=round(ll, 2), min_var=round(mv, 3),
min_var_item=mi, sizes=cnt, hits=0)); u['hits'] += 1
    top = sorted(uniq.values(), key=lambda u: -u['ll'])
    print(f'K = {K}: {len(sols)} converged starts; five highest log-likelihoods:')
    for u in top[:5]:
        print('    ', u, '(spurious)' if u['min_var'] < ADMISSIBLE_MIN_VAR else '')

# Highest-likelihood solutions found with 200-start fits (Table S2, "Highest LL found")
for K, seed in [(4, 102), (5, 101)]:
    g = lpa_fit(X, K, n_starts=200, seed=seed)
    print(f'K = {K}, 200 starts (seed {seed}): LL = {g["ll"]:.2f}; smallest within-profile
variance = {g["var"].min():.3f} (Item {g["var"].argmin() + 1}); '
          f'spurious = {g["var"].min() < ADMISSIBLE_MIN_VAR}')
```

```python
# Best admissible four-profile solution and cross-classification with the three-profile solution
f4 = lpa_fit(X, 4, n_starts=200, seed=100, min_var=ADMISSIBLE_MIN_VAR); o4 =
np.argsort(f4['means'].mean(1)); h4 = np.argsort(o4)[f4['hard']]
assert f4['admissible'] and round(f4['ll'], 2) == -2691.71
r4 = f4['resp']; av4 = [r4[f4['hard'] == k, k].mean() for k in range(4)]
print(f"Admissible K = 4: LL = {f4['ll']:.2f}, AIC = {f4['aic']:.2f}, BIC = {f4['bic']:.2f}, SA-
BIC = {f4['sabic']:.2f}, entropy = {f4['entropy']:.3f}, AvePP = {min(av4):.3f}-{max(av4):.3f}")
print(pd.crosstab(pd.Series(h_main, name='3 profiles'), pd.Series(h4, name='4 profiles')))
f2 = lpa_fit(X, 2, n_starts=50, seed=2, min_var=ADMISSIBLE_MIN_VAR); o2 =
np.argsort(f2['means'].mean(1)); h2 = np.argsort(o2)[f2['hard']]
print(pd.crosstab(pd.Series(h_main, name='3 profiles'), pd.Series(h2, name='2 profiles')))

# Within-profile residual correlations of the three-profile model
resid = X - f3['means'][f3['hard']]; C = np.corrcoef(resid.T); iu = np.triu_indices(11, 1)
print(f'Residual correlations: mean {C[iu].mean():.2f}, max {C[iu].max():.2f}; pairs above .30:
{(np.abs(C[iu]) > .3).sum()} of {len(iu[0])}')
```

```
K = 2: 400 converged starts; five highest log-likelihoods:
    {'ll': -2800.52, 'min_var': 0.374, 'min_var_item': 2, 'sizes': [88, 125], 'hits': 395}
    {'ll': -3036.14, 'min_var': 0.568, 'min_var_item': 9, 'sizes': [1, 212], 'hits': 2}
    {'ll': -3051.25, 'min_var': 0.562, 'min_var_item': 3, 'sizes': [4, 209], 'hits': 2}
    {'ll': -3069.96, 'min_var': 0.617, 'min_var_item': 9, 'sizes': [1, 212], 'hits': 1}
K = 3: 400 converged starts; five highest log-likelihoods:
    {'ll': -2743.24, 'min_var': 0.161, 'min_var_item': 2, 'sizes': [35, 62, 116], 'hits': 194}
    {'ll': -2748.93, 'min_var': 0.3, 'min_var_item': 9, 'sizes': [15, 87, 111], 'hits': 19}
    {'ll': -2750.78, 'min_var': 0.284, 'min_var_item': 7, 'sizes': [24, 73, 116], 'hits': 14}
    {'ll': -2751.72, 'min_var': 0.212, 'min_var_item': 7, 'sizes': [27, 70, 116], 'hits': 6}
    {'ll': -2753.51, 'min_var': 0.341, 'min_var_item': 7, 'sizes': [1, 88, 124], 'hits': 8}
K = 4: 400 converged starts; five highest log-likelihoods:
    {'ll': -2691.71, 'min_var': 0.162, 'min_var_item': 2, 'sizes': [2, 35, 71, 105], 'hits': 5}
    {'ll': -2693.27, 'min_var': 0.16, 'min_var_item': 2, 'sizes': [1, 35, 63, 114], 'hits': 10}
    {'ll': -2694.4, 'min_var': 0.22, 'min_var_item': 1, 'sizes': [15, 16, 79, 103], 'hits': 7}
    {'ll': -2695.55, 'min_var': 0.166, 'min_var_item': 1, 'sizes': [8, 18, 77, 110], 'hits': 5}
    {'ll': -2695.64, 'min_var': 0.279, 'min_var_item': 3, 'sizes': [5, 15, 79, 114], 'hits': 2}
K = 5: 400 converged starts; five highest log-likelihoods:
    {'ll': -2460.65, 'min_var': 0.004, 'min_var_item': 1, 'sizes': [12, 15, 25, 68, 93], 'hits':
1} (spurious)
    {'ll': -2537.66, 'min_var': 0.008, 'min_var_item': 10, 'sizes': [10, 25, 40, 51, 87], 'hits':
2} (spurious)
    {'ll': -2639.14, 'min_var': 0.199, 'min_var_item': 2, 'sizes': [5, 18, 22, 58, 110], 'hits':
8}
    {'ll': -2644.08, 'min_var': 0.225, 'min_var_item': 1, 'sizes': [5, 11, 16, 70, 111], 'hits':
4}
    {'ll': -2644.84, 'min_var': 0.171, 'min_var_item': 1, 'sizes': [1, 10, 15, 79, 108], 'hits':
1}
K = 4, 200 starts (seed 102): LL = -2647.82; smallest within-profile variance = 0.020 (Item 7);
spurious = True
K = 5, 200 starts (seed 101): LL = -2460.65; smallest within-profile variance = 0.004 (Item 1);
spurious = True
Admissible K = 4: LL = -2691.71, AIC = 5499.41, BIC = 5694.37, SA-BIC = 5510.58, entropy = 0.927,
AvePP = 0.940-1.000
4 profiles   0  1   2    3
3 profiles
0           35  0   0    0
1            0  1  61    0
2            0  1  10  105
2 profiles   0    1
3 profiles
0           35    0
1           53    9
```

```
2           0  116
Residual correlations: mean 0.06, max 0.47; pairs above .30: 1 of 55
```

In [15]:

```
# B3. Parametric bootstrap likelihood ratio test (Table S2)
ref = {K: lpa_fit(X, K, n_starts=1 if K == 1 else 100, seed=K, min_var=ADMISSIBLE_MIN_VAR) for K
in [1, 2, 3]}
ref[4] = lpa_fit(X, 4, n_starts=200, seed=100, min_var=ADMISSIBLE_MIN_VAR)
assert all(ref[K]['admissible'] for K in ref)
rng = np.random.default_rng(2026)
for k in [2, 3, 4]:
    LR_obs = 2 * (ref[k]['ll'] - ref[k - 1]['ll']); LRs = []; replaced = no_adm = 0
    conv = {'converged': 0, 'empty': 0, 'max_iter': 0}
    for b in range(200):
        Xb = lpa_simulate(ref[k - 1], n, rng)
        f0 = lpa_fit(Xb, k - 1, n_starts=1 if k - 1 == 1 else 15, seed=b,
min_var=ADMISSIBLE_MIN_VAR)
        f1 = lpa_fit(Xb, k, n_starts=20, seed=10000 + b, min_var=ADMISSIBLE_MIN_VAR)
        add_diag(conv, f0); add_diag(conv, f1)
        replaced += f0['replaced'] + f1['replaced']; no_adm += (not f0['admissible']) + (not
f1['admissible'])
        if f0['admissible'] and f1['admissible']:
            LRs.append(2 * (f1['ll'] - f0['ll']))
    LRs = np.array(LRs); B_ = len(LRs)
    print(f'{k} vs {k - 1}: LR = {LR_obs:.1f}; bootstrap samples used = {B_} of 200; bootstrap p
= {(np.sum(LRs >= LR_obs) + 1) / (B_ + 1):.3f}; '
          f'95th percentile = {np.percentile(LRs, 95):.1f}; max = {LRs.max():.1f}')
    print(f'   starts {conv}; inadmissible highest-likelihood solutions replaced = {replaced};
fits without an admissible solution = {no_adm}')
```

```
2 vs 1: LR = 549.6; bootstrap samples used = 200 of 200; bootstrap p = 0.005; 95th percentile =
31.7; max = 39.1
   starts {'converged': 4200, 'empty': 0, 'max_iter': 0}; inadmissible highest-likelihood
solutions replaced = 0; fits without an admissible solution = 0
3 vs 2: LR = 114.6; bootstrap samples used = 200 of 200; bootstrap p = 0.005; 95th percentile =
32.9; max = 46.5
   starts {'converged': 7000, 'empty': 0, 'max_iter': 0}; inadmissible highest-likelihood
solutions replaced = 0; fits without an admissible solution = 0
4 vs 3: LR = 103.1; bootstrap samples used = 200 of 200; bootstrap p = 0.005; 95th percentile =
32.6; max = 40.0
   starts {'converged': 7000, 'empty': 0, 'max_iter': 0}; inadmissible highest-likelihood
solutions replaced = 0; fits without an admissible solution = 0
```

In [16]:

```
# B4. Alternative specifications (Table S3)
# (a) Latent class models with categorical indicators (class-specific category probabilities)
def lca(Y, C, K, starts=60, seed=0):
    rng = np.random.default_rng(seed); best = None; oh = np.eye(C)[Y]; d = Y.shape[1]
    for _ in range(starts):
        pi = rng.dirichlet(np.ones(K)); th_ = rng.dirichlet(np.ones(C), size=(K, d)); prev = -
np.inf
        for _ in range(3000):
            lj = np.log(pi)[None, :] + np.einsum('ndc,kdc->nk', oh, np.log(th_)); lse =
logsumexp(lj, 1); ll = lse.sum(); r = np.exp(lj - lse[:, None])
            pi = r.mean(0); th_ = np.einsum('nk,ndc->kdc', r, oh) + 1e-6; th_ /= th_.sum(2,
keepdims=True)
            if abs(ll - prev) < 1e-9 * (1 + abs(ll)):
                break
```

```
            prev = ll
        if best is None or ll > best[0]:
            best = (ll, r, th_)
    ll, r, th_ = best; p = K * d * (C - 1) + K - 1; h = r.argmax(1)
    ev = (th_ * np.arange(1, C + 1)[None, None, :]).sum(2); o = np.argsort(ev.mean(1)); ho =
np.argsort(o)[h]
    ent = np.nan if K == 1 else 1 + np.sum(r * np.log(np.maximum(r, 1e-300))) / (len(Y) *
np.log(K))
    return dict(LL=round(ll, 2), p=p, BIC=round(-2 * ll + p * np.log(len(Y)), 2), SABIC=round(-2
* ll + p * np.log((len(Y) + 2) / 24), 2),
                entropy=round(ent, 3), sizes=np.bincount(ho, minlength=K).tolist(), ARI=
(round(ARI(h_main, ho), 3) if K == 3 else None))

for C_, lab in [(4, '4 categories (1 and 2 collapsed)'), (5, '5 categories')]:
    Y = (np.clip(X.astype(int), 2, 5) - 2) if C_ == 4 else (X.astype(int) - 1)
    print(lab)
    for K in range(1, 6):
        print('   K =', K, lca(Y, C_, K, starts=1 if K == 1 else 60, seed=K))

# (b) Residual covariance between Items 6 and 7 (equal across profiles)
blk = [5, 6]; oth = [j for j in range(11) if j not in blk]
def lpa_ld(Xd, K, starts=60, seed=0, min_var=None):
    # min_var: return the admissible solution (every pooled within-profile variance >= min_var)
with the largest LL
    rng = np.random.default_rng(seed); nn, d = Xd.shape; best = None
    for _ in range(starts):
        if K == 1:
            pi = np.ones(1); mu = Xd.mean(0, keepdims=True)
        else:
            idx = rng.choice(nn, K, replace=False); mu = Xd[idx] + rng.normal(0, .05, (K, d));
pi = rng.dirichlet(np.ones(K))
        var = Xd.var(0); S2 = np.cov(Xd[:, blk].T, bias=True); prev = -np.inf
        for _ in range(3000):
            Si = np.linalg.inv(S2); _, ld2 = np.linalg.slogdet(S2); lj = np.zeros((nn, K))
            for k in range(K):
                e = Xd - mu[k]
                lj[:, k] = -0.5 * (np.sum(np.log(2 * np.pi * var[oth])) + np.sum(e[:, oth] ** 2
/ var[oth], 1) + 2 * np.log(2 * np.pi) + ld2
                                   + np.einsum('ij,jk,ik->i', e[:, blk], Si, e[:, blk])) +
np.log(max(pi[k], 1e-300))
            lse = logsumexp(lj, 1); ll = lse.sum(); r = np.exp(lj - lse[:, None]); Nk = r.sum(0)
            if np.any(Nk < 1e-8):
                break
            pi = Nk / nn; mu = (r.T @ Xd) / Nk[:, None]
            var = np.maximum(sum((r[:, k:k + 1] * (Xd - mu[k]) ** 2).sum(0) for k in range(K)) /
nn, 1e-3)
            S2 = sum(((r[:, k:k + 1] * (Xd[:, blk] - mu[k, blk])).T @ (Xd[:, blk] - mu[k, blk]))
for k in range(K)) / nn
            if abs(ll - prev) < 1e-9 * (1 + abs(ll)):
                break
            prev = ll
        ok = min_var is None or var.min() >= min_var
        if ok and (best is None or ll > best[0]):
            best = (ll, r.copy(), mu.copy(), var.copy(), S2.copy(), pi.copy())
    ll, r, mu, var, S2, pi = best; p = K * d + d + 1 + (K - 1); o = np.argsort(mu.mean(1)); h =
np.argsort(o)[r.argmax(1)]
    # independent check of the log-likelihood with scipy (full Gaussian density, normalizing
constants included)
    Sig = np.diag(var.copy()); Sig[np.ix_(blk, blk)] = S2
    ll_check = logsumexp(np.column_stack([np.log(pi[k]) + multivariate_normal(mu[k],
```

```
Sig).logpdf(Xd) for k in range(K)]), axis=1).sum()
    assert abs(ll_check - ll) < 1e-4
    return dict(LL=round(ll, 2), p=p, BIC=round(-2 * ll + p * np.log(nn), 2), AIC=round(-2 * ll
+ 2 * p, 2),
                SABIC=round(-2 * ll + p * np.log((nn + 2) / 24), 2), sizes=np.bincount(h,
minlength=K).tolist(),
                min_var=round(var.min(), 3), ARI=(round(ARI(h_main, h), 3) if K == 3 else None))
print('Residual covariance between Items 6 and 7 (highest-likelihood solution; admissible = all
variances >= 0.10)')
for K in range(1, 5):
    res = lpa_ld(X, K, starts=1 if K == 1 else 60, seed=K)
    print('   K =', K, res, '' if res['min_var'] >= ADMISSIBLE_MIN_VAR else '-> inadmissible')
    if res['min_var'] < ADMISSIBLE_MIN_VAR:
        print('   K =', K, 'best admissible:', lpa_ld(X, K, starts=60, seed=K,
min_var=ADMISSIBLE_MIN_VAR))

# (c) Class-specific variances (lower bound 0.05)
print('Class-specific variances')
for K in range(1, 5):
    g = lpa_fit(X, K, n_starts=1 if K == 1 else 100, seed=K, shared=False, var_floor=0.05)
    print(f"   K = {K}: LL = {g['ll']:.2f}, parameters = {g['p']}, BIC = {g['bic']:.2f}, sizes =
{sorted(g['counts'].tolist())}, "
          f"variances at bound = {int((g['var'] <= .0501).sum())}, admissible =
{bool(g['var'].min() >= ADMISSIBLE_MIN_VAR)}")
print('   (0.05 is the computational lower bound for class-specific variances; variances at this
bound are below 0.10, so these solutions are inadmissible.)')

# (d) Continuous factor models and a factor mixture model
for m in [1, 2]:
    fa = FactorAnalysis(n_components=m).fit(X); ll = fa.score(X) * n; p = 11 * m + 22 - m * (m -
1) // 2
    Sig_fa = fa.components_.T @ fa.components_ + np.diag(fa.noise_variance_)
    ll_check = multivariate_normal(fa.mean_, Sig_fa).logpdf(X).sum(); assert abs(ll_check - ll)
< 1e-4
    print(f'{m}-factor model: LL = {ll:.2f}, parameters = {p}, AIC = {-2 * ll + 2 * p:.2f}, BIC
= {-2 * ll + p * np.log(n):.2f}, SA-BIC = {-2 * ll + p * np.log((n + 2) / 24):.2f}')

# Likelihood conventions of the compared Gaussian models (Table S3, panel C)
ll_main_check = logsumexp(np.column_stack([np.log(f3['pi'][k]) + multivariate_normal(f3['means']
[k], np.diag(f3['var'])).logpdf(X) for k in range(3)]), axis=1).sum()
assert abs(ll_main_check - f3['ll']) < 1e-4
print(f'Comparability: every Gaussian model in panel C was fitted by maximum likelihood to the
same n = {n} respondents and d = {X.shape[1]} indicators (reverse-coded, 1-5); '
      f'each log-likelihood is the sum of the full multivariate normal (mixture) log-densities
including normalizing constants and was re-computed with scipy '
      f'(three-profile model: {ll_main_check:.2f}); BIC = -2 LL + p ln({n}).')
print('Two-factor varimax loadings:'); print(np.round(FactorAnalysis(2,
rotation='varimax').fit(X).components_.T, 2))

def fmm(Xd, K, m=1, starts=25, seed=0, iters=1500):
    rng = np.random.default_rng(seed); nn, d = Xd.shape; best = None
    for _ in range(starts):
        r = np.ones((nn, 1)) if K == 1 else rng.dirichlet(np.ones(K), size=nn); prev = -np.inf
        for it in range(iters):
            Nk = r.sum(0); pi = Nk / nn; mu = (r.T @ Xd) / Nk[:, None]
            S = sum(((r[:, k:k + 1] * (Xd - mu[k])).T @ (Xd - mu[k])) for k in range(K)) / nn
            if it == 0:
                ev, evec = np.linalg.eigh(S); Lm = evec[:, -m:] * np.sqrt(ev[-m:]); Psi =
np.diag(S).copy() * .5
            for _ in range(5):
```

```
                Sig = Lm @ Lm.T + np.diag(Psi); Bm = Lm.T @ np.linalg.inv(Sig); Ezz = np.eye(m) - Bm @ Lm + Bm @ S @ Bm.T
                Lm = S @ Bm.T @ np.linalg.inv(Ezz); Psi = np.maximum(np.diag(S - Lm @ Bm @ S), 1e-3)
            Sig = Lm @ Lm.T + np.diag(Psi); Si = np.linalg.inv(Sig); _, ld = np.linalg.slogdet(Sig)
            lj = np.stack([-0.5 * (d * np.log(2 * np.pi) + ld + np.einsum('ij,jk,ik->i', Xd - mu[k], Si, Xd - mu[k])) for k in range(K)], 1) + np.log(pi)
            lse = logsumexp(lj, 1); ll = lse.sum(); r = np.exp(lj - lse[:, None])
            if abs(ll - prev) < 1e-8 * (1 + abs(ll)):
                break
            prev = ll
        if best is None or ll > best[0]:
            best = (ll, r, mu, Psi.copy(), Lm.copy(), pi.copy())
    ll, r, mu, Psi, Lm, pi = best; p = K * d + (K - 1) + d * m + d - m * (m - 1) // 2
    # independent check of the log-likelihood with scipy (full Gaussian mixture density, normalizing constants included)
    Sig = Lm @ Lm.T + np.diag(Psi)
    ll_check = logsumexp(np.column_stack([np.log(pi[k]) + multivariate_normal(mu[k], Sig).logpdf(Xd) for k in range(K)]), axis=1).sum()
    assert abs(ll_check - ll) < 1e-4
    return ll, p, r, mu, Psi
for K in [2, 3]:
    ll, p, r, mu, Psi = fmm(X, K, starts=25, seed=K)
    print(f'Factor mixture, 1 factor, {K} classes: LL = {ll:.2f}, parameters = {p}, AIC = {-2 * ll + 2 * p:.2f}, BIC = {-2 * ll + p * np.log(n):.2f}, '
          f'SA-BIC = {-2 * ll + p * np.log((n + 2) / 24):.2f}, sizes = {np.bincount(r.argmax(1)).tolist()}, smallest unique variance = {Psi.min():.3f}, '
          f'admissible = {bool(Psi.min() >= ADMISSIBLE_MIN_VAR)}')
    print('   class means:', np.round(mu, 2).tolist())
```

```
4 categories (1 and 2 collapsed)
   K = 1 {'LL': np.float64(-2673.45), 'p': 33, 'BIC': np.float64(5523.82), 'SABIC': np.float64(5419.25), 'entropy': nan, 'sizes': [213], 'ARI': None}
   K = 2 {'LL': np.float64(-2259.3), 'p': 67, 'BIC': np.float64(4877.82), 'SABIC': np.float64(4665.51), 'entropy': np.float64(0.942), 'sizes': [91, 122], 'ARI': None}
   K = 3 {'LL': np.float64(-2156.09), 'p': 101, 'BIC': np.float64(4853.68), 'SABIC': np.float64(4533.64), 'entropy': np.float64(0.941), 'sizes': [71, 84, 58], 'ARI': 0.316}
   K = 4 {'LL': np.float64(-2069.55), 'p': 135, 'BIC': np.float64(4862.87), 'SABIC': np.float64(4435.09), 'entropy': np.float64(0.946), 'sizes': [25, 50, 80, 58], 'ARI': None}
   K = 5 {'LL': np.float64(-2024.61), 'p': 169, 'BIC': np.float64(4955.27), 'SABIC': np.float64(4419.76), 'entropy': np.float64(0.946), 'sizes': [15, 25, 47, 69, 57], 'ARI': None}
5 categories
   K = 1 {'LL': np.float64(-2741.04), 'p': 44, 'BIC': np.float64(5717.97), 'SABIC': np.float64(5578.55), 'entropy': nan, 'sizes': [213], 'ARI': None}
   K = 2 {'LL': np.float64(-2305.13), 'p': 89, 'BIC': np.float64(5087.41), 'SABIC': np.float64(4805.4), 'entropy': np.float64(0.945), 'sizes': [90, 123], 'ARI': None}
   K = 3 {'LL': np.float64(-2182.02), 'p': 134, 'BIC': np.float64(5082.45), 'SABIC': np.float64(4657.85), 'entropy': np.float64(0.949), 'sizes': [71, 84, 58], 'ARI': 0.316}
   K = 4 {'LL': np.float64(-2095.42), 'p': 179, 'BIC': np.float64(5150.51), 'SABIC': np.float64(4583.31), 'entropy': np.float64(0.954), 'sizes': [25, 50, 80, 58], 'ARI': None}
   K = 5 {'LL': np.float64(-2049.93), 'p': 224, 'BIC': np.float64(5300.8), 'SABIC': np.float64(4591.01), 'entropy': np.float64(0.967), 'sizes': [22, 20, 34, 79, 58], 'ARI': None}
Residual covariance between Items 6 and 7 (highest-likelihood solution; admissible = all variances >= 0.10)
   K = 1 {'LL': np.float64(-3013.58), 'p': 23, 'BIC': np.float64(6150.47), 'AIC': np.float64(6073.16), 'SABIC': np.float64(6077.59), 'sizes': [213], 'min_var': np.float64(0.619), 'ARI': None}
   K = 2 {'LL': np.float64(-2770.22), 'p': 35, 'BIC': np.float64(5728.09), 'AIC': np.float64(5610.44), 'SABIC': np.float64(5617.18), 'sizes': [87, 126], 'min_var':
```

```
np.float64(0.367), 'ARI': None}
   K = 3 {'LL': np.float64(-2707.4), 'p': 47, 'BIC': np.float64(5666.78), 'AIC':
np.float64(5508.8), 'SABIC': np.float64(5517.85), 'sizes': [35, 79, 99], 'min_var':
np.float64(0.154), 'ARI': 0.719}
   K = 4 {'LL': np.float64(-2652.04), 'p': 59, 'BIC': np.float64(5620.39), 'AIC':
np.float64(5422.07), 'SABIC': np.float64(5433.44), 'sizes': [35, 70, 32, 76], 'min_var':
np.float64(0.043), 'ARI': None} -> inadmissible
   K = 4 best admissible: {'LL': np.float64(-2654.15), 'p': 59, 'BIC': np.float64(5624.61),
'AIC': np.float64(5426.29), 'SABIC': np.float64(5437.65), 'sizes': [35, 9, 74, 95], 'min_var':
np.float64(0.148), 'ARI': None}
Class-specific variances
   K = 1: LL = -3075.31, parameters = 22, BIC = 6268.57, sizes = [213], variances at bound = 0,
admissible = True
   K = 2: LL = -2526.15, parameters = 45, BIC = 5293.56, sizes = [52, 161], variances at bound =
5, admissible = False
   K = 3: LL = -2336.07, parameters = 68, BIC = 5036.70, sizes = [46, 54, 113], variances at
bound = 9, admissible = False
   K = 4: LL = -2217.96, parameters = 91, BIC = 4923.79, sizes = [42, 49, 50, 72], variances at
bound = 11, admissible = False
   (0.05 is the computational lower bound for class-specific variances; variances at this bound
are below 0.10, so these solutions are inadmissible.)
1-factor model: LL = -2773.31, parameters = 33, AIC = 5612.63, BIC = 5723.55, SA-BIC = 5618.98
2-factor model: LL = -2722.77, parameters = 43, AIC = 5531.53, BIC = 5676.07, SA-BIC = 5539.81
Comparability: every Gaussian model in panel C was fitted by maximum likelihood to the same n =
213 respondents and d = 11 indicators (reverse-coded, 1-5); each log-likelihood is the sum of the
full multivariate normal (mixture) log-densities including normalizing constants and was re-
computed with scipy (three-profile model: -2743.24); BIC = -2 LL + p ln(213).
Two-factor varimax loadings:
[[-0.14  0.48]
 [-0.27  0.6 ]
 [-0.34  0.29]
 [-0.07  0.64]
 [-0.18  0.45]
 [-0.65  0.09]
 [-0.69  0.17]
 [-0.28  0.59]
 [-0.47  0.3 ]
 [-0.41  0.33]
 [-0.41  0.34]]
Factor mixture, 1 factor, 2 classes: LL = -2722.01, parameters = 45, AIC = 5534.02, BIC =
5685.28, SA-BIC = 5542.69, sizes = [172, 41], smallest unique variance = 0.228, admissible = True
   class means: [[4.58, 4.63, 4.5, 3.8, 4.53, 4.39, 4.44, 4.22, 4.45, 4.34, 3.93], [3.55, 2.98,
3.67, 2.69, 3.54, 4.02, 3.79, 3.16, 3.65, 3.61, 3.29]]
Factor mixture, 1 factor, 3 classes: LL = -2618.28, parameters = 57, AIC = 5350.55, BIC =
5542.14, SA-BIC = 5361.53, sizes = [118, 69, 26], smallest unique variance = 0.038, admissible =
False
   class means: [[5.0, 4.65, 4.54, 3.86, 4.62, 4.58, 4.55, 4.42, 4.53, 4.38, 4.06], [3.99, 4.07,
4.14, 3.44, 4.1, 4.04, 4.02, 3.6, 4.05, 4.0, 3.54], [2.51, 3.29, 3.86, 2.68, 3.67, 3.82, 3.94,
3.22, 3.82, 3.82, 3.29]]
```

In [17]:

```python
# B5. Nonparametric bootstrap stability of the three-profile solution (Table S4, panel A)
incons = (raw11.std(1) == 0) | (X[:, 2] == 1)          # identical responses to all items, or
lowest response to Item 3
print('Inconsistent responders (NO):', NO[incons].tolist())
P_items = [0, 1, 2, 3, 4, 7]; S_items = [5, 6, 8, 9, 10]

def summarize(Ms, Ps, As, lab_align):
    low = Ms[:, 0]
```

```
    print(f'  [{lab_align}] median sizes (%): {np.round(100 * np.median(Ps, 0), 1)} | 2.5%: {np.round(100 * np.percentile(Ps, 2.5, 0), 1)} | 97.5%: {np.round(100 * np.percentile(Ps, 97.5, 0), 1)}')
    print(f'  [{lab_align}] High-practice mean item score (median): {np.median(Ms[:, 2].mean(1)):.2f}; '
          f'profile smaller than 5% in {100 * np.mean(Ps.min(1) < .05):.1f}%; lowest profile privacy mean < security mean in {100 * np.mean(low[:, P_items].mean(1) < low[:, S_items].mean(1)):.1f}%')

for lab, Xd in [('Full sample', X), ('Excluding 11 inconsistent responders', X[~incons])]:
    nn = len(Xd); fref = lpa_fit(Xd, 3, n_starts=100, seed=3, min_var=ADMISSIBLE_MIN_VAR); assert fref['admissible']
    oref = np.argsort(fref['means'].mean(1)); href = np.argsort(oref)[fref['hard']]; Mref = fref['means'][oref]
    rng = np.random.default_rng(2024); Ms, Ps, As, Mh, Ph = [], [], [], [], []
    conv = {'converged': 0, 'empty': 0, 'max_iter': 0}; replaced = no_adm = agree = 0
    for b in range(200):
        idx = rng.integers(0, nn, nn); g = lpa_fit(Xd[idx], 3, n_starts=30, seed=b, min_var=ADMISSIBLE_MIN_VAR); add_diag(conv, g)
        if not g['admissible']:
            no_adm += 1; continue
        replaced += g['replaced']
        ob = np.argsort(g['means'].mean(1)); Mb = g['means'][ob]; pib = g['pi'][ob]
        same, cols = matches_reference(Mb, Mref); agree += same
        inv = np.argsort(cols)                                  # Hungarian alignment: reference profile j <- Mb[inv[j]]
        lj = -0.5 * (np.sum(np.log(2 * np.pi * g['var'])) + np.sum((Xd[:, None, :] - Mb[None]) ** 2 / g['var'], 2)) + np.log(pib)
        Ms.append(Mb); Ps.append(pib); As.append(ARI(href, lj.argmax(1))); Mh.append(Mb[inv]); Ph.append(pib[inv])
    Ms, Ps, As, Mh, Ph = map(np.array, (Ms, Ps, As, Mh, Ph)); used = len(As)
    print(f'\n{lab} (n = {nn}); reference sizes {np.bincount(href).tolist()}')
    print(f'  resamples: 200; with an admissible solution (used): {used}; without: {no_adm}; inadmissible highest-likelihood solutions replaced: {replaced}')
    print(f'  starts (30 per resample): {conv}')
    print(f'  alignment by mean item score agreed with Hungarian matching to the reference means in {agree} of {used} resamples')
    summarize(Ms, Ps, As, 'ordered by mean score')
    summarize(Mh, Ph, As, 'Hungarian matching')
    print(f'  ARI median {np.median(As):.2f} (IQR {np.percentile(As, 25):.2f}-{np.percentile(As, 75):.2f}); ARI >= .80 in {100 * np.mean(As >= .8):.1f}%')
```

```
Inconsistent responders (NO): [5, 6, 18, 76, 86, 122, 131, 135, 155, 158, 173]

Full sample (n = 213); reference sizes [35, 62, 116]
  resamples: 200; with an admissible solution (used): 200; without: 0; inadmissible highest-likelihood solutions replaced: 48
  starts (30 per resample): {'converged': 6000, 'empty': 0, 'max_iter': 0}
  alignment by mean item score agreed with Hungarian matching to the reference means in 140 of 200 resamples
  [ordered by mean score] median sizes (%): [14.4 34.4 53.8] | 2.5%: [ 0.9  2.3 39.5] | 97.5%: [44.  46.9 61.1]
  [ordered by mean score] High-practice mean item score (median): 4.59; profile smaller than 5% in 35.0%; lowest profile privacy mean < security mean in 45.5%
  [Hungarian matching] median sizes (%): [22.8 27.7 53.8] | 2.5%: [ 6.4  0.9 39.5] | 97.5%: [46.6 44.1 61.1]
  [Hungarian matching] High-practice mean item score (median): 4.59; profile smaller than 5% in 35.0%; lowest profile privacy mean < security mean in 70.5%
  ARI median 0.70 (IQR 0.65-0.73); ARI >= .80 in 14.0%
```

```
Excluding 11 inconsistent responders (n = 202); reference sizes [29, 58, 115]
  resamples: 200; with an admissible solution (used): 200; without: 0; inadmissible highest-
likelihood solutions replaced: 60
  starts (30 per resample): {'converged': 6000, 'empty': 0, 'max_iter': 0}
  alignment by mean item score agreed with Hungarian matching to the reference means in 156 of
200 resamples
  [ordered by mean score] median sizes (%): [24.  28.1 53.9] | 2.5%: [ 5.   4.5 32.9] | 97.5%:
[43.  43.6 63. ]
  [ordered by mean score] High-practice mean item score (median): 4.61; profile smaller than 5%
in 10.0%; lowest profile privacy mean < security mean in 68.5%
  [Hungarian matching] median sizes (%): [17.1 30.8 53.9] | 2.5%: [ 4.5  5.7 32.9] | 97.5%: [42.5
43.6 63. ]
  [Hungarian matching] High-practice mean item score (median): 4.61; profile smaller than 5% in
10.0%; lowest profile privacy mean < security mean in 87.5%
  ARI median 0.75 (IQR 0.65-0.79); ARI >= .80 in 21.0%
```

In [18]:

```python
# B6. Monte Carlo simulations (Table S4, panels B and C)
f3b = lpa_fit(X, 3, n_starts=100, seed=3, min_var=ADMISSIBLE_MIN_VAR); assert f3b['admissible']

# (a) class enumeration with continuous simulated data (K = 1-4); best admissible solution for
every K
rng = np.random.default_rng(99); pick = {'BIC': [], 'AIC': [], 'SABIC': []}; recov = []
no_adm = {K: 0 for K in range(1, 5)}; repl = {K: 0 for K in range(1, 5)}; conv = {'converged':
0, 'empty': 0, 'max_iter': 0}
for r_ in range(100):
    Xs = lpa_simulate(f3b, n, rng); crit = {'BIC': [], 'AIC': [], 'SABIC': []}
    for K in range(1, 5):
        g = lpa_fit(Xs, K, n_starts=1 if K == 1 else 15, seed=r_ * 10 + K,
min_var=ADMISSIBLE_MIN_VAR); add_diag(conv, g)
        no_adm[K] += not g['admissible']; repl[K] += g['replaced']
        for c in crit:
            crit[c].append(g[c.lower()] if g['admissible'] else np.inf)
        if K == 3 and g['admissible']:
            cost = ((g['means'][:, None, :] - f3b['means'][None]) ** 2).sum(2); a_, c_ =
linear_sum_assignment(cost)
            recov.append(np.abs(g['means'][a_] - f3b['means'][c_]).mean())
    for c in pick:
        pick[c].append(int(np.argmin(crit[c])) + 1)
print('Continuous data, 100 samples:', {c: {K: pick[c].count(K) for K in range(1, 5)} for c in
pick},
      f'| mean absolute error of means = {np.mean(recov):.2f} ({len(recov)} samples)')
print(f'   fits without an admissible solution by K: {no_adm}; inadmissible solutions replaced
by K: {repl}; starts {conv}')

# (b) class enumeration with data rounded to the 1-5 format (K = 1-5)
#     unrestricted: highest-likelihood solution for each K (shows the effect of spurious
solutions);
#     admissible: best admissible solution for each K and every profile >= 5% of the sample
(size rule of the main analysis)
rng = np.random.default_rng(99); pick = {'BIC': [], 'AIC': [], 'SABIC': [], 'BIC admissible':
[]}
no_adm = {K: 0 for K in range(1, 6)}; repl = {K: 0 for K in range(1, 6)}; small = {K: 0 for K in
range(1, 6)}
conv = {'converged': 0, 'empty': 0, 'max_iter': 0}; pick_no3 = []
for r_ in range(100):
    Xs = np.clip(np.rint(lpa_simulate(f3b, n, rng)), 1, 5); crit = {'BIC': [], 'AIC': [],
'SABIC': []}; bic_adm = []
    for K in range(1, 6):
```

```
        g = lpa_fit(Xs, K, n_starts=1 if K == 1 else 15, seed=r_ * 10 + K, min_var=ADMISSIBLE_MIN_VAR); add_diag(conv, g)
        p = g['p']; ll_any = g['ll_any']
        crit['BIC'].append(-2 * ll_any + p * np.log(n)); crit['AIC'].append(-2 * ll_any + 2 * p); crit['SABIC'].append(-2 * ll_any + p * np.log((n + 2) / 24))
        no_adm[K] += not g['admissible']; repl[K] += g['replaced']
        size_ok = g['admissible'] and g['counts'].min() >= .05 * n; small[K] += g['admissible'] and not size_ok
        bic_adm.append(g['bic'] if size_ok else np.inf)
    for c in ['BIC', 'AIC', 'SABIC']:
        pick[c].append(int(np.argmin(crit[c])) + 1)
    pick['BIC admissible'].append(int(np.argmin(bic_adm)) + 1)      # K = 1 is always admissible, so every sample selects a model
    if not np.isfinite(bic_adm[2]):
        pick_no3.append(pick['BIC admissible'][-1])
print('Rounded data, 100 samples:', {c: {K: pick[c].count(K) for K in range(1, 6)} for c in pick})
print("   (BIC, AIC, SABIC: highest-likelihood solution for each K, criterion not applied, shown for comparison; "
      "'BIC admissible': rule used in this study)")
print(f'   fits without an admissible solution by K: {no_adm}; inadmissible solutions replaced by K: {repl}; '
      f'admissible but a profile < 5% by K: {small}; starts {conv}')
print(f'   samples in which no admissible three-profile solution with every profile >= 5% was found: {len(pick_no3)}; '
      f'BIC (admissible) selected K = {dict(sorted({K: pick_no3.count(K) for K in set(pick_no3)}.items()))} in these samples')

# (c) power for the age association, full procedure, data rounded to the 1-5 format
o3b = np.argsort(f3b['means'].mean(1)); R3b = f3b['resp'][:, o3b]; h3b = R3b.argmax(1)
Btrue, _, _ = three_step(Z, h3b, error_matrix(R3b, h3b), se=False, gtol=1e-7, maxiter=5000)
mu_t = f3b['means'][o3b]; var_t = f3b['var']
rng = np.random.default_rng(31); sig_w = sig_b = used = no_adm_p = repl_p = small_p = se_na = 0
for r_ in range(200):
    idx = rng.integers(0, n, n); Zs = Z[idx]
    eta = np.column_stack([Zs @ Btrue.T, np.zeros(n)]); pr = np.exp(eta - logsumexp(eta, 1)[:, None])
    cls = np.array([rng.choice(3, p=pp) for pp in pr])
    Xs = np.clip(np.rint(mu_t[cls] + rng.normal(size=(n, 11)) * np.sqrt(var_t)), 1, 5)
    fs = lpa_fit(Xs, 3, n_starts=20, seed=r_, min_var=ADMISSIBLE_MIN_VAR)
    if not fs['admissible']:
        no_adm_p += 1; continue
    repl_p += fs['replaced']
    os_ = np.argsort(fs['means'].mean(1)); Rs = fs['resp'][:, os_]; hs = Rs.argmax(1)
    if np.bincount(hs, minlength=3).min() < 5:              # same exclusion rule as the bootstrap of the three-step procedure (B8)
        small_p += 1; continue
    used += 1; Ds = error_matrix(Rs, hs)
    Bs, Ss, llf = three_step(Zs, hs, Ds, gtol=1e-7, maxiter=5000, absval=False)
    _, _, ll0 = three_step(np.column_stack([Zs[:, 0], Zs[:, 3:]]), hs, Ds, se=False, gtol=1e-7, maxiter=5000)
    se_na += not np.isfinite(Ss[1, 2])                          # standard error not estimable: counted as non-significant
    sig_w += bool(np.isfinite(Ss[1, 2]) and 2 * norm.sf(abs(Bs[1, 2] / Ss[1, 2])) < .05); sig_b += chi2.sf(2 * (llf - ll0), 4) < .05
print(f'Power: 200 simulated samples; analysed {used}; excluded {no_adm_p} without an admissible solution and {small_p} in which a '
      f'profile had fewer than 5 members; inadmissible highest-likelihood solutions replaced: {repl_p}; '
      f'analysed samples with a non-estimable standard error for age >= 50: {se_na}')
```

```
print(f'   Wald test for age >= 50 (true OR = {np.exp(Btrue[1, 2]):.2f}): significant in {sig_w} 
samples = {sig_w / used:.3f} of the analysed samples '
      f'and {sig_w / 200:.3f} of all 200 samples (excluded samples counted as non-significant)')
print(f'   Likelihood ratio test of the age block (df = 4): significant in {sig_b} samples = 
{sig_b / used:.3f} of the analysed samples '
      f'and {sig_b / 200:.3f} of all 200 samples')
```

```
Continuous data, 100 samples: {'BIC': {1: 0, 2: 0, 3: 100, 4: 0}, 'AIC': {1: 0, 2: 0, 3: 36, 4: 
64}, 'SABIC': {1: 0, 2: 0, 3: 70, 4: 30}} | mean absolute error of means = 0.08 (100 samples)
   fits without an admissible solution by K: {1: 0, 2: 0, 3: 0, 4: 0}; inadmissible solutions 
replaced by K: {1: 0, 2: 0, 3: 0, 4: 0}; starts {'converged': 4600, 'empty': 0, 'max_iter': 0}
Rounded data, 100 samples: {'BIC': {1: 0, 2: 0, 3: 15, 4: 21, 5: 64}, 'AIC': {1: 0, 2: 0, 3: 0, 
4: 4, 5: 96}, 'SABIC': {1: 0, 2: 0, 3: 0, 4: 5, 5: 95}, 'BIC admissible': {1: 0, 2: 6, 3: 92, 4: 
2, 5: 0}}
   (BIC, AIC, SABIC: highest-likelihood solution for each K, criterion not applied, shown for 
comparison; 'BIC admissible': rule used in this study)
   fits without an admissible solution by K: {1: 0, 2: 0, 3: 6, 4: 4, 5: 6}; inadmissible 
solutions replaced by K: {1: 0, 2: 0, 3: 2, 4: 63, 5: 80}; admissible but a profile < 5% by K: 
{1: 0, 2: 0, 3: 0, 4: 33, 5: 54}; starts {'converged': 6100, 'empty': 0, 'max_iter': 0}
   samples in which no admissible three-profile solution with every profile >= 5% was found: 6; 
BIC (admissible) selected K = {2: 4, 4: 2} in these samples
Power: 200 simulated samples; analysed 187; excluded 9 without an admissible solution and 4 in 
which a profile had fewer than 5 members; inadmissible highest-likelihood solutions replaced: 10; 
analysed samples with a non-estimable standard error for age >= 50: 0
   Wald test for age >= 50 (true OR = 2.89): significant in 125 samples = 0.668 of the analysed 
samples and 0.625 of all 200 samples (excluded samples counted as non-significant)
   Likelihood ratio test of the age block (df = 4): significant in 139 samples = 0.743 of the 
analysed samples and 0.695 of all 200 samples
```

In [19]:

```
# B7. Sensitivity analyses (Table S5)
def sens(lab, Xd, rows):
    f, o, R_, h_, D_ = ordered_fit(Xd, 150, 3)
    a_ = age_code[rows]; oc = occ_code[rows]; mm = len(rows)
    Zd = np.column_stack([np.ones(mm), (a_ == 3) * 1., (a_ >= 4) * 1., (oc == 1) * 1., 
np.isin(oc, [2, 9]) * 1., np.isin(oc, [5, 6, 7, 8]) * 1.])
    keepc = [0] + [j for j in range(1, Zd.shape[1]) if Zd[:, j].std() > 0]; Zd = Zd[:, keepc]
    Bs, Ss, llf = three_step(Zd, h_, D_)
    _, _, ll0 = three_step(np.column_stack([Zd[:, 0], Zd[:, 3:]]), h_, D_, se=False)
    assert f['admissible'], lab
    bics = []
    for K in range(2, 5):
        g = lpa_fit(Xd, K, n_starts=100, seed=K, min_var=ADMISSIBLE_MIN_VAR)
        note = '' if g['admissible'] else ', no admissible solution'
        note += f", replaced spurious LL {g['ll_any']:.1f}" if g['replaced'] else ''
        bics.append(f"{g['bic']:.1f} ({int(g['counts'].min())}{note})")
    print(f"{lab}: n = {mm}, sizes = {np.bincount(h_, minlength=3).tolist()}, ARI = 
{ARI(h_main[rows], h_):.3f}, three-profile solution replaced = {f['replaced']}, BIC K=2/3/4 
(smallest) = {bics}, "
          f"age>=50 OR = {np.exp(Bs[1, 2]):.2f} ({np.exp(Bs[1, 2] - 1.96 * Ss[1, 2]):.2f}-
{np.exp(Bs[1, 2] + 1.96 * Ss[1, 2]):.2f}), "
          f"p = {2 * norm.sf(abs(Bs[1, 2] / Ss[1, 2])):.3f}, age block p = {chi2.sf(2 * (llf - 
ll0), 4):.3f}")

clin = ~np.isin(occ_code, [2, 9]); ex4 = np.isin(NO, [6, 131, 135, 173])
X12 = np.column_stack([X, 6 - df['PIP_13'].astype(float).to_numpy()])
sens('Main analysis', X, np.arange(n))
sens('Clinical staff only', X[clin], np.where(clin)[0])
sens('Excluding the 4 respondents excluded in the earlier study', X[~ex4], np.where(~ex4)[0])
```

```
sens('Excluding 11 inconsistent responders', X[~incons], np.where(~incons)[0])
sens('Adding screened-out item P3 (12 indicators)', X12, np.arange(n))

# Registered nurses (code 3) and nursing assistants (code 4) as separate categories
f, o, R_, h_, D_ = ordered_fit(X, 100, 3); assert f['admissible'] and not f['replaced']
print(pd.crosstab(np.select([occ_code == 3, occ_code == 4], ['RN', 'NA'], 'other'), h_))
Z5 = np.column_stack([np.ones(n), age40, age50, phys, (occ_code == 4) * 1., admin, other])
Bs, Ss, llf = three_step(Z5, h_, D_)
names5 = ['Age 40-49', 'Age >=50', 'Physician', 'NA (vs RN)', 'Admin/support', 'Other health']
for c, lab in enumerate(['Low', 'Moderate']):
    for j, nm in enumerate(names5, start=1):
        print(f'  {lab} vs High | {nm:13s} OR {np.exp(Bs[c, j]):.2f} ({np.exp(Bs[c, j] - 1.96 *
Ss[c, j]):.2f}-{np.exp(Bs[c, j] + 1.96 * Ss[c, j]):.2f}) p {2 * norm.sf(abs(Bs[c, j] / Ss[c,
j])):.3f}')
_, _, ll_na = three_step(np.column_stack([np.ones(n), age40, age50, phys, admin, other]), h_,
D_, se=False)
_, _, ll_no = three_step(np.column_stack([np.ones(n), age40, age50]), h_, D_, se=False)
print(f'RN vs NA: LR = {2 * (llf - ll_na):.2f}, df = 2, p = {chi2.sf(2 * (llf - ll_na), 2):.3f};
occupation block (5 categories): LR = {2 * (llf - ll_no):.2f}, df = 8, p = {chi2.sf(2 * (llf -
ll_no), 8):.3f}')
```

```
Main analysis: n = 213, sizes = [35, 62, 116], ARI = 1.000, three-profile solution replaced =
False, BIC K=2/3/4 (smallest) = ['5783.3 (88)', '5733.1 (35)', '5694.4 (2)'], age>=50 OR = 2.89
(1.25-6.70), p = 0.013, age block p = 0.008
Clinical staff only: n = 170, sizes = [27, 52, 91], ARI = 0.959, three-profile solution replaced
= False, BIC K=2/3/4 (smallest) = ['4651.2 (75)', '4611.0 (27)', '4591.0 (9)'], age>=50 OR = 3.60
(1.39-9.30), p = 0.008, age block p = 0.000
Excluding the 4 respondents excluded in the earlier study: n = 209, sizes = [31, 61, 117], ARI =
0.983, three-profile solution replaced = False, BIC K=2/3/4 (smallest) = ['5663.3 (86)', '5625.3
(31)', '5587.0 (2)'], age>=50 OR = 2.75 (1.19-6.36), p = 0.018, age block p = 0.045
Excluding 11 inconsistent responders: n = 202, sizes = [29, 58, 115], ARI = 0.965, three-profile
solution replaced = False, BIC K=2/3/4 (smallest) = ['5229.9 (85)', '5192.7 (29)', '5169.0 (17,
replaced spurious LL -2352.8)'], age>=50 OR = 2.80 (1.22-6.44), p = 0.015, age block p = 0.032
Adding screened-out item P3 (12 indicators): n = 213, sizes = [35, 61, 117], ARI = 0.984, three-
profile solution replaced = False, BIC K=2/3/4 (smallest) = ['6399.9 (91)', '6353.2 (35)',
'6296.8 (11)'], age>=50 OR = 2.70 (1.18-6.13), p = 0.018, age block p = 0.010
col_0   0   1   2
row_0
NA      8  19  16
RN     16  17  39
other  11  26  61
  Low vs High | Age 40-49     OR 2.30 (0.84-6.26) p 0.104
  Low vs High | Age >=50      OR 0.70 (0.25-1.99) p 0.508
  Low vs High | Physician     OR 0.18 (0.02-1.69) p 0.132
  Low vs High | NA (vs RN)    OR 1.05 (0.34-3.24) p 0.938
  Low vs High | Admin/support OR 0.65 (0.23-1.85) p 0.422
  Low vs High | Other health  OR 0.17 (0.03-0.90) p 0.037
  Moderate vs High | Age 40-49     OR 1.65 (0.60-4.55) p 0.330
  Moderate vs High | Age >=50      OR 2.60 (1.10-6.14) p 0.029
  Moderate vs High | Physician     OR 1.46 (0.41-5.18) p 0.559
  Moderate vs High | NA (vs RN)    OR 2.57 (0.95-6.93) p 0.062
  Moderate vs High | Admin/support OR 1.02 (0.36-2.89) p 0.974
  Moderate vs High | Other health  OR 1.23 (0.43-3.55) p 0.703
RN vs NA: LR = 3.98, df = 2, p = 0.137; occupation block (5 categories): LR = 13.61, df = 8, p =
0.092
```

In [20]:

```
# B8. Employment-duration model and bootstrap of the full three-step procedure (Table S6)
f, o, R_, h_, D_ = ordered_fit(X, 100, 3); assert f['admissible'] and not f['replaced']
```

```
Zt = np.column_stack([np.ones(n), ten520, ten20, phys, admin, other])
Bt, St, llt = three_step(Zt, h_, D_)
for c, lab in enumerate(['Low', 'Moderate']):
    for j, nm in enumerate(['Employment 5-<20', 'Employment >=20', 'Physician', 'Admin/support', 
'Other health'], start=1):
        print(f'  {lab} vs High | {nm:17s} OR {np.exp(Bt[c, j]):.2f} ({np.exp(Bt[c, j] - 1.96 * 
St[c, j]):.2f}-{np.exp(Bt[c, j] + 1.96 * St[c, j]):.2f}) p {2 * norm.sf(abs(Bt[c, j] / St[c, 
j])):.3f}')
_, _, ll0 = three_step(np.ones((n, 1)), h_, D_, se=False)
_, _, llt_no = three_step(np.column_stack([np.ones(n), phys, admin, other]), h_, D_, se=False)
print(f'Overall LR = {2 * (llt - ll0):.2f}, df = 10, p = {chi2.sf(2 * (llt - ll0), 10):.3f}; 
employment block LR = {2 * (llt - llt_no):.2f}, '
      f'df = 4, p = {chi2.sf(2 * (llt - llt_no), 4):.3f}; McFadden R2 = {1 - llt / ll0:.3f}')

M_ref = f['means'][o]
rng = np.random.default_rng(11); boots = []; boots_h = []
no_adm = replaced = too_small = agree = 0; conv = {'converged': 0, 'empty': 0, 'max_iter': 0}
for b in range(200):
    idx = rng.integers(0, n, n)
    fb, ob, Rb, hb, Db = ordered_fit(X[idx], 20, b); add_diag(conv, fb)
    if not fb['admissible']:
        no_adm += 1; continue
    replaced += fb['replaced']
    if np.bincount(hb, minlength=3).min() < 5:
        too_small += 1; continue
    same, cols = matches_reference(fb['means'][ob], M_ref); agree += same
    Bb, _, _ = three_step(Z[idx], hb, Db, se=False, gtol=1e-7, maxiter=5000); boots.append(Bb[:, 
1:])
    if not same:                                          # same resample with Hungarian-
aligned labels
        perm = np.argsort(cols); Rh = Rb[:, perm]; hh = Rh.argmax(1)
        if np.bincount(hh, minlength=3).min() >= 5:
            Bh, _, _ = three_step(Z[idx], hh, error_matrix(Rh, hh), se=False, gtol=1e-7, 
maxiter=5000); boots_h.append(Bh[:, 1:])
        else:
            boots_h.append(None)
    else:
        boots_h.append(Bb[:, 1:])
boots = np.array(boots)
print(f'Resamples: 200; without an admissible solution: {no_adm}; inadmissible highest-
likelihood solutions replaced: {replaced}; '
      f'excluded because a profile had fewer than 5 members: {too_small}; used: {len(boots)}')
print(f'Starts (20 per resample): {conv}; ordering by mean item score agreed with Hungarian 
matching in {agree} of {len(boots)} used resamples')
for c, lab in enumerate(['Low', 'Moderate']):
    for j, nm in enumerate(['Age 40-49', 'Age >=50', 'Physician', 'Admin/support', 'Other 
health']):
        lo, md_, hi = np.percentile(boots[:, c, j], [2.5, 50, 97.5])
        print(f'  {lab} vs High | {nm:13s} median OR {np.exp(md_):.2f} (95% interval 
{np.exp(lo):.2f}-{np.exp(hi):.2f}); OR > 1 in {100 * np.mean(boots[:, c, j] > 0):.0f}%')
bh = np.array([x for x in boots_h if x is not None])
lo, md_, hi = np.percentile(bh[:, 1, 1], [2.5, 50, 97.5])
print(f'Hungarian-aligned labels ({len(bh)} resamples): age >= 50, Moderate vs High: median OR 
{np.exp(md_):.2f} (95% interval {np.exp(lo):.2f}-{np.exp(hi):.2f})')
```

```
  Low vs High | Employment 5-<20  OR 1.09 (0.43-2.79) p 0.852
  Low vs High | Employment >=20   OR 0.44 (0.14-1.45) p 0.178
  Low vs High | Physician         OR 0.13 (0.01-1.33) p 0.085
  Low vs High | Admin/support     OR 0.59 (0.22-1.57) p 0.288
  Low vs High | Other health      OR 0.15 (0.03-0.78) p 0.025
```

```
  Moderate vs High | Employment 5-<20  OR 0.71 (0.29-1.73) p 0.447
  Moderate vs High | Employment >=20   OR 1.79 (0.73-4.43) p 0.205
  Moderate vs High | Physician         OR 0.86 (0.26-2.84) p 0.800
  Moderate vs High | Admin/support     OR 0.75 (0.29-1.99) p 0.568
  Moderate vs High | Other health      OR 0.84 (0.30-2.34) p 0.739
Overall LR = 20.75, df = 10, p = 0.023; employment block LR = 10.93, df = 4, p = 0.027; McFadden
R2 = 0.049
Resamples: 200; without an admissible solution: 0; inadmissible highest-likelihood solutions
replaced: 46; excluded because a profile had fewer than 5 members: 40; used: 160
Starts (20 per resample): {'converged': 4000, 'empty': 0, 'max_iter': 0}; ordering by mean item
score agreed with Hungarian matching in 142 of 160 used resamples
  Low vs High | Age 40-49     median OR 2.51 (95% interval 0.00-9.53); OR > 1 in 91%
  Low vs High | Age >=50      median OR 1.06 (95% interval 0.11-4.84); OR > 1 in 54%
  Low vs High | Physician     median OR 0.43 (95% interval 0.00-3.16); OR > 1 in 24%
  Low vs High | Admin/support median OR 0.53 (95% interval 0.07-1.45); OR > 1 in 14%
  Low vs High | Other health  median OR 0.26 (95% interval 0.00-1.62); OR > 1 in 10%
  Moderate vs High | Age 40-49     median OR 1.77 (95% interval 0.00-25.04); OR > 1 in 79%
  Moderate vs High | Age >=50      median OR 1.91 (95% interval 0.42-14.39); OR > 1 in 87%
  Moderate vs High | Physician     median OR 0.41 (95% interval 0.00-3.36); OR > 1 in 26%
  Moderate vs High | Admin/support median OR 0.67 (95% interval 0.00-10.29); OR > 1 in 22%
  Moderate vs High | Other health  median OR 0.62 (95% interval 0.00-3.47); OR > 1 in 25%
Hungarian-aligned labels (160 resamples): age >= 50, Moderate vs High: median OR 1.87 (95%
interval 0.45-7.62)
```

In [21]:

```
# B9. Classification error matrix and validation of the implementation (Table S7)
print('Classification error matrix, P(assigned | latent):'); print(pd.DataFrame(D, index=labels,
columns=labels).round(3))
for eps in [1e-3, 1e-4, 1e-5]:
    H = approx_hess(full['beta'], lambda b: -negll_3step(b, Z, hard3, D), epsilon=eps); s =
np.sqrt(np.diag(-np.linalg.inv(H))).reshape(2, -1)
    print(f'Hessian step {eps:g}: age >= 50 (Moderate vs High) 95% CI {np.exp(B[1, 2] - 1.96 *
s[1, 2]):.2f}-{np.exp(B[1, 2] + 1.96 * s[1, 2]):.2f}')
rng = np.random.default_rng(5)
for N_ in [213, 5000]:
    Xs = lpa_simulate(f3b, N_, rng)
    own = lpa_fit(Xs, 3, n_starts=30, seed=1, shared=False, var_floor=1e-6)
    gm = GaussianMixture(3, covariance_type='diag', n_init=30, random_state=0, reg_covar=1e-9,
tol=1e-10, max_iter=5000).fit(Xs)
    print(f'N = {N_}: EM log-likelihood {own["ll"]:.4f} vs scikit-learn {gm.score(Xs) * N_:.4f};
ARI = {ARI(own["hard"], gm.predict(Xs)):.3f}')
    eei = lpa_fit(Xs, 3, n_starts=30, seed=1); oe = np.argsort(eei['means'].mean(1)); ot =
np.argsort(f3b['means'].mean(1))
    err = np.abs(eei['means'][oe] - f3b['means'][ot])
    print(f'   recovery of profile means: mean absolute error {err.mean():.3f} (max
{err.max():.3f})')
```

```
Classification error matrix, P(assigned | latent):
                   Low-practice  Moderate-practice  High-practice
Low-practice              0.994              0.016          0.000
Moderate-practice         0.006              0.927          0.023
High-practice             0.000              0.057          0.977
Hessian step 0.001: age >= 50 (Moderate vs High) 95% CI 1.25-6.70
Hessian step 0.0001: age >= 50 (Moderate vs High) 95% CI 1.25-6.70
Hessian step 1e-05: age >= 50 (Moderate vs High) 95% CI 1.25-6.70
N = 213: EM log-likelihood -2703.0869 vs scikit-learn -2703.0868; ARI = 1.000
   recovery of profile means: mean absolute error 0.095 (max 0.289)
N = 5000: EM log-likelihood -64263.4110 vs scikit-learn -64263.4110; ARI = 1.000
   recovery of profile means: mean absolute error 0.016 (max 0.046)
```
