# Supplementary material for: Latent Profiles of Self-Reported Patient Privacy Protection Practices Among Hospital Healthcare Workers: Implications for Patient Safety and Interprofessional Education
Source: Healthcare (Basel). 2026 Oct 26;14(21):3623. doi: 10.3390/healthcare14213623 (PMC13762769; doi:10.3390/healthcare14213623)
Supplement: Supplementary file 1 [file healthcare-14-03623-s001.zip › Supplementary_Tables_S1-S7_updated.pdf]

Supplementary Materials

Latent Profiles of Self-Reported Patient Privacy Protection Practices among Hospital Healthcare Workers: Implications for Patient Safety and Interprofessional Education. All values were computed from the item responses (N = 213) with the code in Supplementary File S1.

Admissibility criterion. In every analysis that re-estimated a latent profile model (Tables S2–S6), a solution was classified as spurious (inadmissible) when the pooled within-profile variance of any indicator was below 0.10, and the admissible solution with the largest log-likelihood across starting values was retained; fits without an admissible solution were excluded and counted. The numbers of converged starts, replaced solutions and usable replications are reported in the table notes and printed in File S1.

Table S1. Rasch-based screening of the practice items

Eleven of the 18 practice items were retained. Fit statistics are from the Rasch rating scale model calibrated on 202 respondents, after excluding the 11 respondents with the poorest person fit.

Item	Practice item (English version published in [23])	Keying	Measure (SE)	Infit MnSq (Z)	Outfit MnSq (Z)	Decision
P1	I collect as much personal information as possible during the registration and consultation process.	−	1.01 (0.09)	1.55 (4.9)	1.76 (6.2)	Removed
P2	I have used patient personal information, collected for medical purposes, for non-medical purposes such as writing research papers or promotional activities.	−	−0.25 (0.11)	1.73 (5.2)	1.78 (4.8)	Removed
P3	I have accessed other patients' medical records without their consent for reference in a medical context.	−	0.40 (0.09)	1.37 (3.2)	1.39 (3.2)	Removed[a]
P4	I have accessed medical records and test results created by other departments, even when unrelated to my job responsibilities.	−	−0.38 (0.11)	0.98 (−0.1)	1.00 (0.0)	Retained (Item 1)
P5	I take precautions to ensure that conversations regarding patient status, such as during handover procedures, cannot be overheard by unauthorized individuals.	+	−0.49 (0.11)	0.59 (−4.0)	0.77 (−1.7)	Removed
P6	I have discussed patient status with employees who are not directly involved in the patient's treatment.	−	−0.16 (0.10)	0.78 (−2.0)	0.72 (−2.3)	Retained (Item 2)
P7	I avoid sensitive discussions with patients in shared rooms and, if necessary, conduct consultations in a private setting.	+	−0.43 (0.11)	0.86 (−1.2)	0.81 (−1.4)	Retained (Item 3)
P8	When referring a patient to another department or medical institution for consultation, I provide as much personal information as possible.	−	0.99 (0.09)	1.02 (0.2)	1.14 (1.3)	Retained (Item 4)
P9	I have provided information regarding a patient's hospitalization or treatment status to their acquaintance.	−	−0.25 (0.11)	1.00 (0.0)	1.04 (0.4)	Retained (Item 5)
P10	I have logged into the medical information system using another employee's ID, either upon their request or with their permission.	−	0.01 (0.10)	1.44 (3.5)	1.34 (2.5)	Removed
P11	I regularly change my password for the medical information system.	+	−0.30 (0.11)	1.07 (0.6)	1.25 (1.8)	Retained (Item 6)
P12	I log out after completing my use of the medical information system or when leaving my workstation for an extended period.	+	−0.23 (0.11)	0.84 (−1.4)	0.99 (0.0)	Retained (Item 7)

Item	Practice item (English version published in [23])	Keying	Measure (SE)	Infit MnSq (Z)	Outfit MnSq (Z)	Decision
P13	I have shared my medical information system ID and password with colleagues in my department.	−	0.43 (0.09)	1.05 (0.5)	0.99 (0.0)	Retained (Item 8)
P14	I store paper charts, photographs, and other medical records in lockable cabinets or secure storage.	+	−0.24 (0.11)	0.63 (−3.6)	0.76 (−2.0)	Retained (Item 9)
P15	I regularly participate in personal information protection training.	+	−0.60 (0.12)	0.51 (−4.9)	0.50 (−4.1)	Removed
P16	Personal information protection training is helpful in ensuring the secure management of medical information.	+	−0.27 (0.11)	0.53 (−4.9)	0.56 (−3.9)	Removed
P17	I immediately dispose of notes related to patients, written for handover or consultation purposes, after work is completed.	+	0.00 (0.10)	0.80 (−1.9)	0.82 (−1.5)	Retained (Item 10)
P18	I either dispose of medical records once the legal retention period expires or request the responsible department to do so.	+	0.76 (0.09)	1.24 (2.2)	1.45 (3.8)	Retained (Item 11)

Summary for the 11 retained items (n = 202)	Value
Person reliability (separation)	.82 (2.14)
Item reliability (separation)	.96 (5.03)
Andrich thresholds (logits)	−2.33, −0.37, 0.37, 2.33
Category use, 1 to 5 (%)	0.5, 5.6, 14.7, 36.5, 42.7
Category average measures, 1 to 5 (logits)	0.97, 0.62, 0.71, 1.63, 3.63
Category outfit MnSq, 1 to 5	12.55, 4.27, 1.30, 0.50, 0.62
Item infit MnSq range	0.63–1.25
Person reliability (separation), all 213 respondents	.79 (1.97)

Notes. Responses were rated on a 5-point scale (1 = strongly disagree to 5 = strongly agree) [23]; negatively keyed items (−) were reverse-scored, so higher scores indicate better practice. Model: Rasch rating scale model [36] estimated by joint maximum likelihood; respondents with extreme scores were excluded from item calibration. Person fit was assessed first, and the 11 respondents with the largest person infit mean-squares (3.32 or higher; the next value was 2.99) were excluded before item fit was evaluated [24]. Removal criterion: infit mean-square outside 0.6–1.4 with |Z| > 2.0. The two lowest categories were rarely used and fitted poorly, and their average measures were not ordered, although the thresholds were ordered; latent class models with these categories collapsed are reported in Table S3.

[a] The original screening (jMetrik 4.1.1) estimated an infit mean-square of 1.48 (Z = 4.07) for P3, and the item was removed. In this Python replication, the estimate was 1.37 (Z = 3.2), just below the cut-off; for the other six removed items, the replication matched the original infit and outfit estimates to within 0.01. Adding P3 as a twelfth indicator reproduced the three-profile solution (ARI = .98; Table S5).

Table S2. Model enumeration diagnostics and bootstrap likelihood ratio tests

The best log-likelihood was replicated for one to three profiles, and the higher-likelihood four- and five-profile solutions were spurious. The BLRT favored each additional profile up to four.

K	Log-likelihood	Parameters	AIC	BIC	SA-BIC	Entropy	Smallest profile, n (%)	Best LL replicated (of 400 starts)	Highest LL found	BLRT vs K − 1: LR (p)
1	−3075.31	22	6194.62	6268.57	6198.86	–	213 (100.0)	Unique solution	–	–
2	−2800.52	34	5669.05	5783.33	5675.59	.900	88 (41.3)	395	Same	549.6 (< .01)
3	−2743.24	46	5578.48	5733.10	5587.34	.915	35 (16.4)	194	Same	114.6 (< .01)
4	−2691.71	58	5499.41	5694.37	5510.58	.927	2 (0.9)	5	−2647.82, spurious (Item 7 variance 0.02)	103.1 (< .01)
5	−2639.14	70	5418.28	5653.57	5431.76	.947	5 (2.3)	8	−2460.65, spurious (Item 1 variance 0.004)	Not computed

Notes. Gaussian latent profile model with profile-specific means, indicator variances equal across profiles and zero within-profile covariances. Rows show the admissible solution with the largest log-likelihood. Each model was estimated from 50 (K = 2–3) or 80 (K = 4–5) initial solutions and re-estimated from 400 random single starts. A solution was classified as spurious when the pooled within-profile variance of any indicator fell below 0.10; the spurious solutions formed profiles around exact response values (McLachlan and Peel [22]). BLRT: parametric bootstrap with 200 samples per comparison [30]; p = (number of bootstrap statistics ≥ observed + 1)/201 = .005 for all three comparisons. The bootstrap 95th percentiles were 31.7 (2 vs 1), 32.9 (3 vs 2) and 32.6 (4 vs 3). In each bootstrap sample, the K − 1 and K profile models were re-estimated from 15 starts (one start for a single profile) and 20 starts, respectively, and the admissible solution with the largest log-likelihood was retained; all starts converged, no highest-likelihood solution was inadmissible, and all 200 samples were used in each comparison.

Cross-classification. The two-profile solution placed all 35 Low-practice respondents and 53 of the 62 Moderate-practice respondents in its lower profile. The admissible four-profile solution reproduced the Low-practice profile exactly (35 respondents), formed a profile of 2 respondents (1 Moderate-practice, 1 High-practice), and moved 10 High-practice respondents into the profile corresponding to Moderate-practice.

Local dependence. In the three-profile solution, within-profile residual correlations averaged .06; the largest was .47 (Items 6 and 7), the only pair of 55 above .30.

Table S3. Alternative model specifications

Ordinal latent class models also had the lowest BIC at three classes. Among Gaussian models fitted to the same data, a two-factor model fitted better than the reported three-profile model, whereas a three-profile model with one post hoc residual covariance fitted slightly better than the two-factor model. Class-specific variances produced inadmissible boundary solutions.

A. Latent class models with categorical indicators

Model	K	Log-likelihood	Parameters	BIC	SA-BIC	Entropy	Class sizes, low to high
4 categories (1 and 2 collapsed)	1	−2673.45	33	5523.82	5419.25	–	213
4 categories (1 and 2 collapsed)	2	−2259.30	67	4877.82	4665.51	.942	91, 122
4 categories (1 and 2 collapsed)	3	−2156.09	101	4853.68	4533.64	.941	71, 84, 58
4 categories (1 and 2 collapsed)	4	−2069.55	135	4862.87	4435.09	.946	25, 50, 80, 58
4 categories (1 and 2 collapsed)	5	−2024.61	169	4955.27	4419.76	.946	15, 25, 47, 69, 57

Model	K	Log-likelihood	Parameters	BIC	SA-BIC	Entropy	Class sizes, low to high
5 categories	1	−2741.04	44	5717.97	5578.55	–	213
5 categories	2	−2305.13	89	5087.41	4805.40	.945	90, 123
5 categories	3	−2182.02	134	5082.45	4657.85	.949	71, 84, 58
5 categories	4	−2095.42	179	5150.51	4583.31	.954	25, 50, 80, 58
5 categories	5	−2049.93	224	5300.80	4591.01	.967	22, 20, 34, 79, 58

Notes. Class-specific category probabilities [32], which place no distributional assumption on the indicators. In both versions, the three-class solution differed mainly in overall level, and its agreement with the latent profile classification was ARI = .32 [34]. Because their likelihood is defined for the discrete responses, their BIC values are not comparable with those of the Gaussian models in panels B and C.

B. Latent profile models with relaxed covariance assumptions

Model	K	Log-likelihood	Parameters	BIC	Profile sizes	Smallest variance	ARI with main solution
Residual covariance, Items 6–7	1	−3013.58	23	6150.47	213	0.62	–
Residual covariance, Items 6–7	2	−2770.22	35	5728.09	87, 126	0.37	–
Residual covariance, Items 6–7	3	−2707.40	47	5666.78	35, 79, 99	0.15	.72
Residual covariance, Items 6–7	4	−2654.15	59	5624.61	35, 9, 74, 95	0.15	–
Class-specific variances	2	−2526.15	45	5293.56	52, 161	5 variances at bound (inadmissible)	–
Class-specific variances	3	−2336.07	68	5036.70	46, 54, 113	9 variances at bound (inadmissible)	–
Class-specific variances	4	−2217.96	91	4923.79	42, 49, 50, 72	11 variances at bound (inadmissible)	–

Notes. Items 6 and 7 were the only indicator pair with a within-profile residual correlation above .30 (Table S2); the residual covariance was therefore a post hoc modification. Rows show the admissible solution with the largest log-likelihood across 60 starts. For four profiles with the residual covariance, the highest-likelihood solution (log-likelihood −2652.04; BIC 5620.39) was inadmissible (smallest variance 0.04) and was replaced; the admissible solution shown has a profile of 9 respondents (4.2%). With class-specific variances, variances converged to the lower bound of 0.05, which was imposed for computation, in every model with two or more profiles; because this bound is below the 0.10 criterion, these boundary solutions were inadmissible and were not interpreted.

C. Continuous measurement models compared with profile models

Model	Log-likelihood	Parameters	AIC	BIC	SA-BIC
One-factor model	−2773.31	33	5612.63	5723.55	5618.98
Two-factor model	−2722.77	43	5531.53	5676.07	5539.81
Factor mixture model, 1 factor and 2 classes	−2722.01	45	5534.02	5685.28	5542.69
Three-profile model (main)	−2743.24	46	5578.48	5733.10	5587.34
Three-profile model with residual covariance, Items 6–7	−2707.40	47	5508.80	5666.78	5517.85

Notes. All models in this panel were fitted by maximum likelihood to the same 213 respondents and the same 11 reverse-coded indicators (1–5 scale) under a multivariate normal likelihood. Each log-likelihood is the sum of the full multivariate normal (or normal mixture) log-densities, including all normalizing constants, and was recomputed independently

with SciPy; BIC = −2 × log-likelihood + p × ln(213), where p counts all free parameters. Exploratory factor models were estimated by maximum likelihood. In the two-factor model, the five reverse-scored privacy items (Items 1, 2, 4, 5 and 8) loaded on one factor (.45–.64) and the security items on the other (.41–.69), whereas Item 3, the only positively worded privacy item, loaded weakly on both (.34 and .29); because item content and wording direction coincide, the two factors may partly reflect item wording. The residual covariance between Items 6 and 7 was added post hoc (panel B). Factor mixture model: class-specific intercepts with a common one-factor covariance structure [33]; its smaller class (n = 41) had particularly low means on Items 2, 4 and 8, and its smallest unique variance was 0.23. A three-class factor mixture solution was spurious (unique variance of Item 1 = 0.04, with classes formed by exact Item 1 values) and is not shown.

Table S4. Resampling stability and Monte Carlo simulations

The High-practice profile was stable across resamples, whereas the division of the remaining respondents into two profiles was not. Simulations showed that the three-profile structure can be recovered at N = 213 when spurious solutions are excluded, and that power for the age association was modest.

A. Nonparametric bootstrap (200 resamples)

Statistic	Full sample (n = 213)	Excluding 11 inconsistent responders (n = 202)
Reference profile sizes: Low, Moderate, High	35, 62, 116	29, 58, 115
Resamples with an admissible three-profile solution (used)	200 of 200	200 of 200
Highest-likelihood solution inadmissible and replaced, resamples	48	60
Ordering by mean score identical to optimal matching, resamples	140 (70.0%)	156 (78.0%)
Low-practice size, median % (95% interval), ordered by mean score	14.4 (0.9–44.0)	24.0 (5.0–43.0)
Low-practice size, optimal matching	22.8 (6.4–46.6)	17.1 (4.5–42.5)
Moderate-practice size, median % (95% interval), ordered by mean score	34.4 (2.3–46.9)	28.1 (4.5–43.6)
Moderate-practice size, optimal matching	27.7 (0.9–44.1)	30.8 (5.7–43.6)
High-practice size, median % (95% interval), either labeling	53.8 (39.5–61.1)	53.9 (32.9–63.0)
High-practice mean item score, median	4.59	4.61
ARI with reference classification, median (IQR)	.70 (.65–.73)	.75 (.65–.79)
Resamples with ARI ≥ .80	14.0%	21.0%
Resamples with a profile smaller than 5%	35.0%	10.0%
Lowest profile with privacy mean below security mean, ordered by mean score	45.5%	68.5%
Same, profile matched to the reference Low-practice profile	70.5%	87.5%

Notes. In each resample, the three-profile model was re-estimated from 30 starts, and the admissible solution with the largest log-likelihood was retained; all 6,000 starts converged in each analysis. Profiles were labeled by ordering them by mean item score, as in the main analysis; as a check, they were also matched to the reference profiles by optimal (Hungarian) matching of profile means. Both labelings gave identical results for the High-practice profile, but the results for the two lower profiles depended on the labeling. ARI compares the reference classification with the classification of the original respondents under each bootstrap solution and does not depend on the labeling. The 11 inconsistent responders were the 7 respondents with identical responses to all 11 items and the 5 respondents who gave the lowest response to Item 3 (one respondent met both conditions).

B. Monte Carlo class enumeration (100 samples of N = 213 generated from the fitted three-profile model)

Generated data	Criterion	K = 1	K = 2	K = 3	K = 4	K = 5
Continuous	BIC	0%	0%	100%	0%	–
Continuous	SA-BIC	0%	0%	70%	30%	–
Continuous	AIC	0%	0%	36%	64%	–
Rounded to the 1–5 format	BIC, criterion not applied	0%	0%	15%	21%	64%
Rounded to the 1–5 format	SA-BIC, criterion not applied	0%	0%	0%	5%	95%
Rounded to the 1–5 format	AIC, criterion not applied	0%	0%	0%	4%	96%
Rounded to the 1–5 format	BIC, admissible solutions (rule used in this study)	0%	6%	92%	2%	0%

Notes. Each cell gives the percentage of the 100 simulated samples (N = 213, generated from the fitted three-profile model) in which that number of profiles had the lowest criterion value, so every row has a denominator of 100 samples; the one-profile model is always admissible, so a model was selected in every sample. Models with one to four (continuous data) or one to five profiles (rounded data) were fitted from 15 starts (one start for K = 1). Continuous data: the admissibility criterion was applied to every fit; all 4,600 starts converged, and every fit had an admissible solution. Rounded data: all 6,100 starts converged. Rows marked “criterion not applied” use the highest-likelihood solution for each K and are shown only to illustrate the effect of spurious solutions. In the row used in this study, each K was represented by its admissible solution with the largest log-likelihood (all within-profile variances ≥ 0.10), and solutions with a profile smaller than 5% of the sample were not eligible. No admissible solution was found in 6, 4 and 6 samples for K = 3, 4 and 5; the highest-likelihood solution was inadmissible and was replaced in 2, 63 and 80 samples; and admissible solutions had a profile smaller than 5% in 33 and 54 samples for K = 4 and 5. In the 6 samples without an eligible three-profile solution, BIC selected two profiles in 4 and four profiles in 2. With continuous data, profile means were recovered with a mean absolute error of 0.08 points.

C. Monte Carlo power for the covariate model (200 samples of N = 213, responses rounded to the 1–5 format)

Test	Power: all 200 samples (analyzed samples, n = 187)
Wald test, age ≥ 50 vs < 40 for Moderate- vs High-practice (true OR = 2.89)	62.5% (66.8%)
Likelihood ratio test of the age block (df = 4)	69.5% (74.3%)

Notes. In each of 200 simulated samples (N = 213), covariates were resampled from the observed data, profile membership was generated from the fitted three-step model, item responses were generated from the profile model and rounded to the 1–5 format, and the full procedure (profile estimation, classification error correction and three-step regression) was repeated. The profile model was fitted from 20 starts, and the admissible solution with the largest log-likelihood was retained (the highest-likelihood solution was replaced in 10 samples). Samples without an admissible solution (9) and samples in which a profile had fewer than five members (4) were not analyzed; they are counted as non-significant in the first value and excluded from the second. Treating the observed odds ratio as the true effect is optimistic, so these values are upper estimates.

Table S5. Sensitivity analyses

The three-profile solution and the direction and size of the age association were similar in every sensitivity analysis.

A. Profile solution and age association

Analysis	n	Profile sizes: Low, Moderate, High	ARI with main solution	BIC for K = 2 / 3 / 4 (smallest K = 4 profile)	Age ≥ 50 vs < 40, Moderate vs High: OR (95% CI), p	Age block p
Main analysis	213	35, 62, 116	–	5783.3 / 5733.1 / 5694.4 (2)	2.89 (1.25–6.70), .013	.008
Clinical staff only	170	27, 52, 91	.96	4651.2 / 4611.0 / 4591.0 (9)	3.60 (1.39–9.30), .008	< .001
Excluding the 4 respondents excluded in [23]	209	31, 61, 117	.98	5663.3 / 5625.3 / 5587.0 (2)	2.75 (1.19–6.36), .018	.045
Excluding 11 inconsistent responders	202	29, 58, 115	.97	5229.9 / 5192.7 / 5169.0 (17)	2.80 (1.22–6.44), .015	.032
Adding screened-out item P3 (12 indicators)	213	35, 61, 117	.98	6399.9 / 6353.2 / 6296.8 (11)	2.70 (1.18–6.13), .018	.010

Notes. Clinical staff: physicians, nursing staff and other healthcare professionals (administrative and support staff excluded). Inconsistent responders: identical responses to all 11 items (n = 7) or the lowest response to Item 3 (n = 5), which may not apply to non-clinical roles; one respondent met both conditions. The four-profile solutions separated small groups: 2 respondents with atypical patterns (main analysis and excluding 4 respondents); 9 and 11 respondents with very low scores on Item 1 (clinical staff only and 12 indicators); and 17 respondents in the sample excluding inconsistent responders, in which the highest-likelihood four-profile solution was spurious (variance of Item 9 = 0.01) and was replaced by the best admissible solution. BIC values are for the admissible solution with the largest log-likelihood across 100 starts. Regression models adjusted for occupation, with nursing staff as the reference.

B. Registered nurses (RNs) and nursing assistants (NAs) as separate categories

Group	n	Low-practice, n (%)	Moderate-practice, n (%)	High-practice, n (%)
RNs	72	16 (22.2)	17 (23.6)	39 (54.2)
NAs	43	8 (18.6)	19 (44.2)	16 (37.2)

Three-step model with RNs as the reference	Estimate
NAs vs RNs, Low- vs High-practice: OR (95% CI), p	1.05 (0.34–3.24), .938
NAs vs RNs, Moderate- vs High-practice: OR (95% CI), p	2.57 (0.95–6.93), .062
Age ≥ 50 vs < 40, Moderate- vs High-practice: OR (95% CI), p	2.60 (1.10–6.14), .029
Separating RNs and NAs: likelihood ratio test	χ^2 = 3.98, df = 2, p = .137
Occupation block with five categories: likelihood ratio test	χ^2 = 13.61, df = 8, p = .092

Notes. Classification error-corrected three-step model with age and occupation (physicians, RNs, NAs, administrative/support staff, other healthcare professionals).

Table S6. Employment-duration model and bootstrap intervals for the main regression model

No individual employment-duration contrast was significant, and the bootstrap intervals for the main model, which include profile-estimation uncertainty, were wide.

A. Three-step model with employment duration in place of age

Outcome (vs High-practice)	Predictor	OR (95% CI)	p
Low-practice	Employment 5 to < 20 years (vs < 5)	1.09 (0.43–2.79)	.852
Low-practice	Employment ≥ 20 years (vs < 5)	0.44 (0.14–1.45)	.178

Outcome (vs High-practice)	Predictor	OR (95% CI)	p
Low-practice	Physician	0.13 (0.01–1.33)	.085
Low-practice	Administrative/support	0.59 (0.22–1.57)	.288
Low-practice	Other healthcare professionals	0.15 (0.03–0.78)	.025
Moderate-practice	Employment 5 to < 20 years (vs < 5)	0.71 (0.29–1.73)	.447
Moderate-practice	Employment ≥ 20 years (vs < 5)	1.79 (0.73–4.43)	.205
Moderate-practice	Physician	0.86 (0.26–2.84)	.800
Moderate-practice	Administrative/support	0.75 (0.29–1.99)	.568
Moderate-practice	Other healthcare professionals	0.84 (0.30–2.34)	.739

Notes. Reference categories: less than 5 years of employment and nursing staff. Overall model: LR $\chi^2 = 20.75$, df = 10, $p = .023$; McFadden pseudo-$R^2 = .049$. Employment-duration block: LR $\chi^2 = 10.93$, df = 4, $p = .027$. Age and employment duration were strongly correlated (Spearman $\rho = .786$), so they were not entered together.

B. Bootstrap of the full three-step procedure for the main model (age and occupation)

Outcome (vs High-practice)	Predictor	OR (Wald 95% CI), Table 5	Bootstrap median OR (95% percentile interval)	Resamples with OR > 1
Low-practice	Age 40–49	2.26 (0.86–5.95)	2.51 (< 0.01–9.53)	91%
Low-practice	Age ≥ 50	0.69 (0.25–1.95)	1.06 (0.11–4.84)	54%
Low-practice	Physician	0.17 (0.02–1.67)	0.43 (< 0.01–3.16)	24%
Low-practice	Administrative/support	0.65 (0.24–1.74)	0.53 (0.07–1.45)	14%
Low-practice	Other healthcare professionals	0.17 (0.03–0.87)	0.26 (< 0.01–1.62)	10%
Moderate-practice	Age 40–49	2.00 (0.75–5.34)	1.77 (< 0.01–25.04)	79%
Moderate-practice	Age ≥ 50	2.89 (1.25–6.70)	1.91 (0.42–14.39)	87%
Moderate-practice	Physician	1.04 (0.31–3.49)	0.41 (< 0.01–3.36)	26%
Moderate-practice	Administrative/support	0.69 (0.27–1.80)	0.67 (< 0.01–10.29)	22%
Moderate-practice	Other healthcare professionals	0.85 (0.32–2.25)	0.62 (< 0.01–3.47)	25%

Notes. In each of 200 nonparametric resamples, the three-profile model was re-estimated from 20 starts, and the admissible solution with the largest log-likelihood was retained; profiles were ordered by mean item score, the classification error matrix was recomputed, and the three-step model was refitted. All starts converged. Every resample had an admissible solution, and in 46 the highest-likelihood solution was inadmissible and was replaced. Resamples in which a profile had fewer than five members (40) were excluded, leaving 160 resamples. Ordering by mean item score agreed with optimal (Hungarian) matching to the reference profile means in 142 of the 160 resamples; with matched labels (160 resamples), the age ≥ 50 odds ratio for Moderate- vs High-practice had a median of 1.87 (95% interval 0.45–7.62). Lower limits below 0.01 reflect complete separation in sparse cells (e.g., only one physician in the Low-practice profile).

Table S7. Classification error matrix, estimation settings, and validation

Classification under the fitted three-profile model was accurate for the Low- and High-practice profiles and somewhat less so for the Moderate-practice profile. The implementation reproduced reference software and recovered known parameters.

A. Classification error matrix, P(assigned profile | latent profile)

Assigned profile	Latent Low-practice	Latent Moderate-practice	Latent High-practice
Low-practice	.994	.016	.000
Moderate-practice	.006	.927	.023
High-practice	.000	.057	.977

Notes. Columns sum to 1. Average posterior probabilities: Low-practice .971, Moderate-practice .955, High-practice .969; entropy = .915. This matrix was held fixed in the third step of the three-step procedure [27,28].

B. Estimation settings and random seeds

Setting	Value
Profile model	Gaussian mixture; profile-specific means; indicator variances equal across profiles; zero within-profile covariances
Estimation	EM; convergence when the change in log-likelihood < 10^{-9} × (1 + |log-likelihood|); maximum 3,000 iterations
Starting values	One start from quantiles of the total score plus random starts (50 in total for K = 2–3; 80 for K = 4–5); 400 additional random single starts for Table S2
Admissibility	Pooled within-profile variance of every indicator ≥ 0.10; applied in every fit (model enumeration, bootstrap likelihood ratio tests, bootstrap resampling, simulations, alternative specifications and sensitivity analyses), retaining the admissible solution with the largest log-likelihood across starts
Convergence	Every start in the bootstrap likelihood ratio tests, the nonparametric bootstrap, the bootstrap of the three-step procedure and the class-enumeration simulations converged within 3,000 iterations (counts in File S1)
Bootstrap profile labels	Ordered by mean item score, as in the main analysis; checked against optimal (Hungarian) matching of profile means to the reference solution (Tables S4 and S6)
Three-step regression	Maximum likelihood with fixed classification error matrix; BFGS optimization; standard errors from the numerical Hessian; Wald confidence intervals
Random seeds	Profile models 1234 + K; random single starts 10000 × K + start; BLRT 2026; nonparametric bootstrap 2024; bootstrap of the three-step procedure 11; Monte Carlo class enumeration 99; Monte Carlo power 31
Software	Original analyses: Python 3.14.6 (NumPy, pandas, SciPy, statsmodels, Matplotlib). Outputs saved in File S1: Python 3.13.16, NumPy 2.5.3, SciPy 1.18.1, pandas 3.0.5, scikit-learn 1.9.1, Matplotlib 3.11.2

C. Validation of the implementation

Check	Result
Independent re-implementation of the selected model and the three-step regression	Identical log-likelihood (−2743.24), profile sizes, item means and Table 5 estimates
Same EM routine with class-specific variances vs scikit-learn GaussianMixture, simulated data (N = 213)	Log-likelihood −2703.0869 vs −2703.0868; ARI = 1.000
Same comparison, simulated data (N = 5,000)	Log-likelihood −64,263.4110 in both; ARI = 1.000
Recovery of profile means from data simulated with the fitted model (N = 213)	Mean absolute error 0.095 (maximum 0.289)
Same, N = 5,000	Mean absolute error 0.016 (maximum 0.046)

Notes. scikit-learn does not offer variances constrained equal across profiles, so the comparison used class-specific diagonal variances. The numerical Hessian for the main regression model gave identical standard errors with step sizes of 10^{-3}, 10^{-4} and 10^{-5}; the sensitivity models used a step of 10^{-4}.
